# Estrogen promotes tumor phenotypes in ER^+^ and ER^–^ breast cancer through *UGDH-GPR30*

**DOI:** 10.1172/jci.insight.197357

**Published:** 2026-09-08

**Authors:** Meghan J. Price, Annee D. Nguyen, Corinne H. Strawser, Trupti Trivedi, Cesar C.D. Baeta, Catherine Lavau, Jovita K. Byemerwa, Debarati Mukherjee, Suzanne E. Wardell, Sandeep Artham, Vardhman Kumar, Shyni Varghese, C. Rory. Goodwin

**Affiliations:** 1 Department of Neurosurgery, Duke University Medical Center, Durham, North Carolina, USA.; 2 Department of Pharmacology and Cancer Biology, Duke University School of Medicine, Durham, North Carolina, USA.; 3 Department of Orthopedics, Duke University Medical Center, Durham, North Carolina, USA.

**Keywords:** Endocrinology, Oncology, Breast cancer, G protein-coupled receptors, Oncogenes

## Abstract

Estrogen can promote aggressive tumor phenotypes in estrogen receptor–positive (ER^+^) breast cancer; however, ER^–^ cell lines are not widely considered estrogen responsive. Noncanonical estrogen-stimulated pathways such as the membrane-bound G protein–coupled estrogen receptor (*GPR30*) can mediate migratory and proliferative phenotypes in breast cancer and are postulated to promote resistance to aromatase therapies. Moreover, dysregulation of UDP-glucose 6-dehydrogenase (*UGDH*), a ubiquitously expressed enzyme critical to the metabolism of UDP-glucuronic acid into extracellular matrix precursors and hormone regulation, is associated with tumorigenesis. Here, we illustrated the impact of estrogen stimulation on tumor phenotypes in ER^+^ and ER^–^ cell models in vitro and in vivo. We then demonstrated *UGDH*’s association with metastatic breast cancer via single-cell sequencing of patient specimens. Genetic knockdown of *UGDH* blunted estrogen-stimulated tumor phenotypes in vitro, ex vivo, and in vivo using both ER^+^ and ER^–^ breast cancer lines. Finally, we demonstrated that *UGDH* knockdown blunted noncanonical estrogen stimulation through *GPR30*. Ultimately, our study validated prior studies demonstrating estrogen-responsive malignant phenotypes in ER^–^ breast cancer and demonstrated that estrogen-stimulated breast cancer progression can be mediated through noncanonical pathways (e.g., *UGDH/GPR30*), regardless of ER status.

## Abstract 2

Estrogen-stimulated breast cancer phenotypes are blunted by genetic knockdown of UGDH in vitro, ex vivo and in vivo, irrespective of estrogen receptor status.

## Introduction

Breast cancer is the most frequently diagnosed cancer globally, accounting for 12% of new cancer diagnoses annually (1, 2). In the United States, breast cancer is responsible for the most cancer-related deaths after lung cancer (2), with the leading cause of death being sequelae of metastatic disease. Although current therapies for breast cancer metastases include a combination of systemic therapies, targeted radiation therapy, and palliative surgery when appropriate, about 20%–30% of ER^+^ breast cancers are intrinsically resistant or become resistant to endocrine therapies (e.g., tamoxifen), resulting in a 30% recurrence rate (3). Survival for metastatic breast cancer is low, with a 5-year survival rate of 27% and 10.81% for patients with estrogen receptor–positive (ER^+^) (1, 4) and ER^–^ disease (5), respectively. Thus, continued efforts to treat and prevent the metastatic spread of both ER^+^ and ER^–^ breast cancer are critical.

Estrogens are responsible for more aggressive phenotypes in ER^+^ breast cancer and, in some cases, ER^–^ breast cancers (3, 6). In ER^+^ breast cancers, estrogens bind the ER in the cell cytosol, after which the ER dimerizes and enters the nucleus to upregulate genes responsible for proliferation and migration. Beyond the canonical ERs, estrogens are shown to interact with non-ER pathways, such as *IGF1R* and the membrane-bound G protein–coupled ER (*GPER1*) (7–13), to mediate migratory and proliferative phenotypes in ER^+^ breast cancer and promote resistance to tamoxifen and other aromatase therapies (14–19). In ER^–^ breast cancer models, 17β-estradiol (E2) is shown to promote more aggressive phenotypes and regulate estrogen-dependent growth factor gene expression, though the mechanism has not been fully elucidated (6).

Several studies have implicated dysregulation of the enzyme UDP-glucose 6-dehydrogenase (*UGDH*) in malignant tumor progression (20–24). *UGDH* is shown to increase the migratory and invasive properties of ovarian cancer (22) and triple-negative breast cancer (8) both in vivo and in vitro, along with increasing stabilization of epithelial-mesenchymal transition–associated (EMT-associated) transcription factors in lung cancer (20, 24). Mechanistically, *UGDH* produces UDP-α-d-glucuronic acid, the precursor for glycosaminoglycans and proteoglycans of the extracellular matrix, and hyaluronic acids (25–27), which are involved in tumor progression and have been noted in high concentrations in a model of ER^–^ breast cancer (3, 6, 28, 29). Separately, *UGDH* is involved in the glucuronidation pathway (30), which regulates hormone processing. Although the role of *UGDH* in migratory pathways is well established, and *UGDH* levels are shown to be responsive to hormones such as E2 (31) in both epithelial and mesenchymal cell lines (32, 33), its impact on estrogen-mediated tumor phenotypes is less well established.

Thus, we sought to assess the role of *UGDH* on estrogen-induced tumorigenic phenotypes in ER^+^ and ER^–^ breast cancer in vitro and in vivo. More specifically, we assessed whether genetic knockdown (KD) of *UGDH* inhibits estrogen-stimulated migratory and invasive phenotypes in vitro and in vivo tumor growth and animal survival in ER^+^ and ER^–^ breast cancer. We also utilized single-cell RNA-seq to determine whether our findings translate to patients with breast cancer using clinical specimens and patient databases to evaluate whether *UGDH* expression is associated with metastatic breast cancer progression.

## Results

### UGDH in breast cancer: clinical expression.

**Figure 1:**
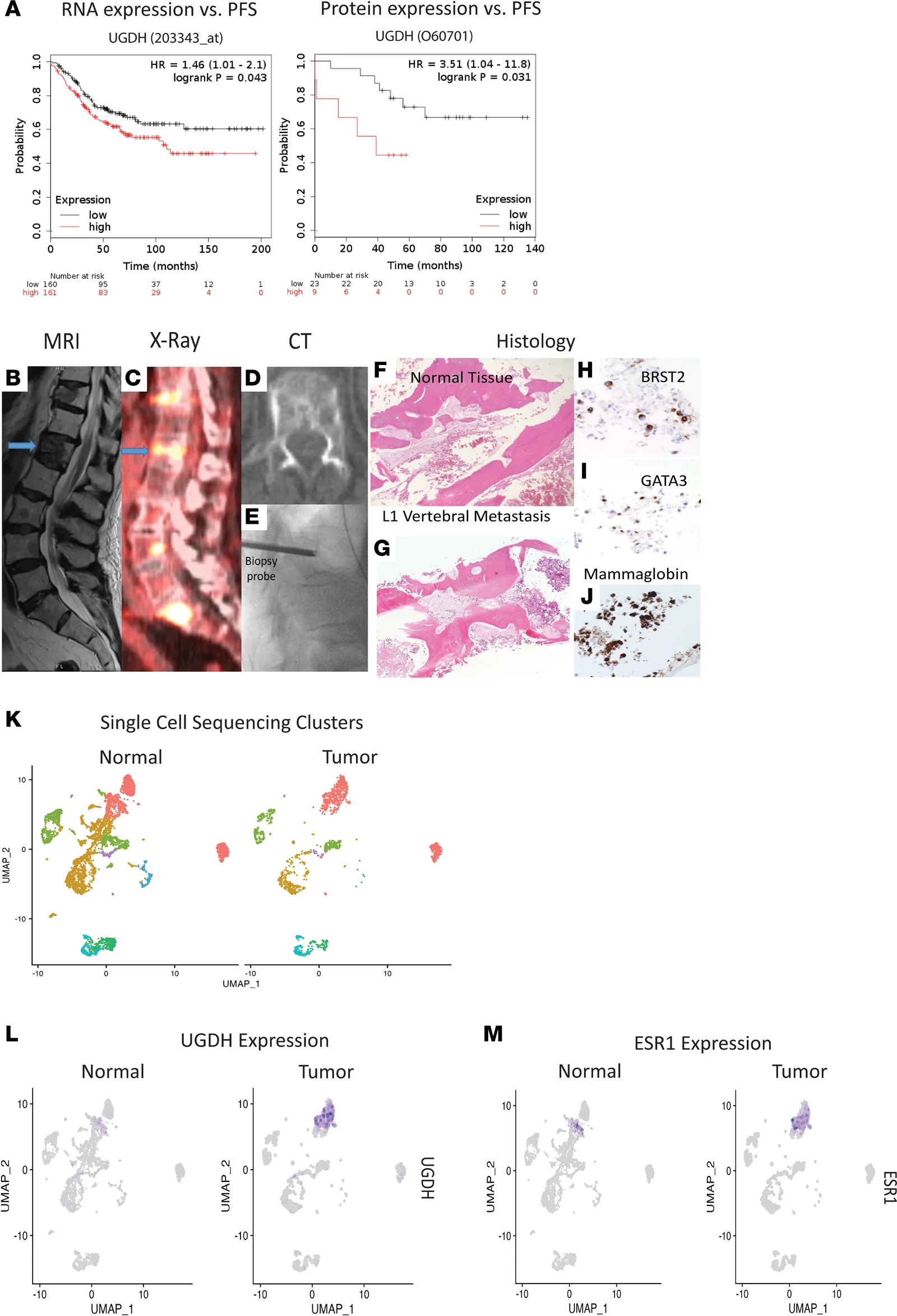
UGDH as a prognostication marker for patient survival and patients with breast cancer metastasis. (**A**) RNA and protein expression of *UGDH* on progression-free survival (PFS). (**B**) Sagittal MRI, (**C**) sagittal x-ray, and (**D**) axial CT scans of patient’s spine, with blue arrows and (**E**) biopsy probe indicating a T7 metastatic lesion. (**F**) H&E histology of normal tissue and (**G**) L1 vertebral metastasis. (**H**) IHC of *BRST2*, (**I**) *GATA3*, and (**J**) mammaglobin confirm that the vertebral metastasis is breast in origin. (**K**) Single-cell sequencing clusters of normal and tumor specimens. (**L**) *UGDH* expression and (**M**) *ESR1* expression in normal and tumor specimens.

To determine whether *UGDH* expression is associated with progression-free survival (PFS) in patients with ER^+^ breast cancer, the online Kaplan-Meier plotter (29) was assessed. Higher *UGDH* RNA and protein expression were associated with worse prognoses for high-grade ER^+^ breast cancer (*P* = 0.043; *P* = 0.0031, respectively). (Figure 1A). To further assess the association of *UGDH* and ER^+^ metastatic breast cancer, clinical specimens obtained from patients with ER^+^ breast cancer with spine metastasis and adjacent normal tissue were obtained for single-cell sequencing analysis. Approximately 70% of patients with advanced breast cancer develop bone metastasis, with the spine being the most common site (34, 35). Moreover, recent studies indicate that breast cancer has a metastatic tropism to the spinal vertebral body (36). After identifying a metastatic L1 lesion through MRI, x-ray, and CT (Figure 1, B–D), we compared the histology of the adjacent normal tissue with the tumor via H&E (Figure 1, F and G). Positive stains of *BRST2*, *GATA3*, and mammaglobin confirmed the osseous metastasis was breast in origin (Figure 1, H–J). Immune profiling results revealed 26 unique clusters of subpopulations across both tissue types, each with their respective sets of differentially expressed genes (DEGs) (Figure 1K). *UGDH* appeared more concentrated in cell clusters in tumor tissue compared with normal vertebrae (Figure 1L), overlapping with *ESR1* expression in similar clusters (Figure 1M).

ER^+^ and ER^–^ breast cancer cell lines were then assessed for baseline *UGDH*. The MCF7:WS8 and T47D (ER^+^) cell lines expressed the highest and lowest levels of *UGDH*, respectively (Supplemental Figure 1, A and B; supplemental material available online with this article; https://doi.org/10.1172/jci.insight.197357DS1), and the MDA-MB-231 and MDA-MB-468 (ER^–^) cell lines had the lowest and second highest expression, respectively (Supplemental Figure 1C). Based on these expression profiles, subsequent in vitro and in vivo analyses were performed with the MCF7:WS8 cell line for ER^+^ and MDA-MB-231 for ER^–^ and replicated with the T47D (ER^+^) and MDA-MB-468 (ER^–^) for reproducibility.

### Estrogen-induced migratory phenotypes are abrogated by UGDH KD in ER^+^ and ER^–^ breast cancer in vitro.

**Figure 2:**
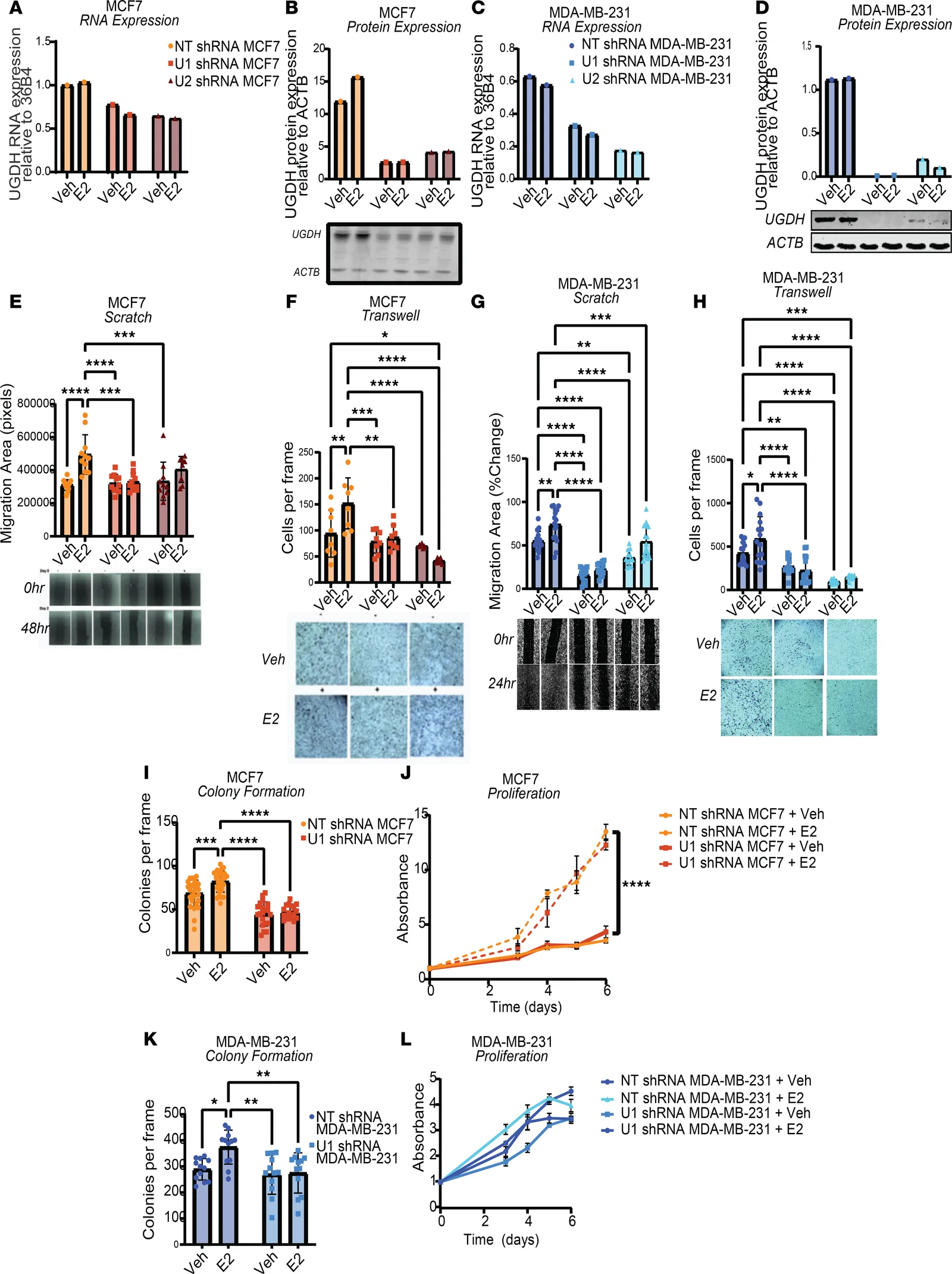
Estrogen-induced migratory phenotypes are abrogated by UGDH KD in ER^+^ and ER^–^ breast cancer in vitro. *UGDH* RNA and protein expression of NT shRNA, U1 shRNA, and U2 shRNA constructs treated with vehicle or E2 for 24 hours in MCF7 cells (**A** and **B**) and MDA-MB-231 (**C** and **D**). (**E**) Differences in wound healing area over 48 hours, with or without E2, in MCF7. (**F**) Number of MCF7 cells migrating in Transwell assay over 16 hours, with or without E2 stimulation, to 10% CFS. Images at 10× original magnification. (**G**) Differences in wound healing area over 48 hours, with or without E2, in MDA-MB-231. (**H**) Number of MDA-MB-231 cells migrating in Transwell assay over 16 hours, with or without E2 stimulation, to 10% CFS. (**I**) Colonies of MCF7 formed per frame, treated with or without E2. (**J**) Proliferation curves of MCF7 treated with or without E2 from day 0 to day 6. (**K**) Colonies of MDA-MB-231 formed per frame, treated with or without E2. (**L**) Proliferation curves of MDA-MB-231 treated with or without E2 from day 0 to day 6. One-way ANOVA; *P < 0.05; **P < 0.01; ***P < 0.001; ****P < 0.0001.

ER^+^ (MCF7:WS8 and T47D) and ER^–^ (MDA-MB-231 and MDA-MB-468) cell lines stably expressing a nontargeting (NT) control shRNA and 2 *UGDH* KD shRNAs (U1 and U2) were generated to assess the effects of decreased *UGDH* expression in ER^+^ and ER^–^ breast cancer cells in vitro (Figure 2, A–D, and Supplemental Figure 2, A and B). In the ER^+^ MCF7:WS8 line, the U1 shRNA decreased *UGDH* RNA (by 30% and 40%, respectively) and protein expression (by 78% and 83%, respectively) in the absence and presence of E2, and the U2 shRNA effectively decreased *UGDH* RNA (by 36% and 39%, respectively) and protein expression (~65% and 73%, respectively) in the absence and presence of E2 (Figure 2, A and B). In MDA-MB-231, the U1 shRNA effectively decreased *UGDH* RNA (by 48% and 53%, respectively) and protein expression (by 99% and 98%, respectively) in the absence and presence of E2; the U2 shRNA effectively decreased *UGDH* RNA (by 72% and 71%, respectively) and protein expression (by 82% and 91%, respectively) in the absence and presence of E2 (Figure 2, C and D). E2 increased wound healing cell migration, Transwell migration, cell colony formation, and cell proliferation phenotypes in the ER^+^ MCF7:WS8 and ER^–^ MDA-MB-231 cell lines in vitro (*P* < 0.001, Figure 2, E–L). *UGDH* KD significantly decreased E2-stimulated wound healing and Transwell migration phenotypes in both ER^+^ (*P* < 0.01, Figure 2, E and F) and ER^–^ cell lines (*P* < 0.01, Figure 2, E and F) in vitro. Additionally, *UGDH* KD altered estrogen-induced colony formation but not proliferation of MCF7 and MDA-MB-231 in vitro (*P* < 0.001, Figure 2, I and J; *P* < 0.01, Figure 2, K and L). Abrogation of estrogen-induced migratory phenotypes by *UGDH* shRNAs was also reproducible in secondary cell lines T47D and MDA-MB-468 (Supplemental Figure 2, C and D, *P* < 0.0001; Supplemental Figure 2E, *P* < 0.001; Supplemental Figure 2F, *P* < 0.01).

### Estrogen promotes tumor growth in breast cancer models in vivo irrespective of ER status, and UGDH KD blunts tumor growth and improves animal survival.

**Figure 3:**
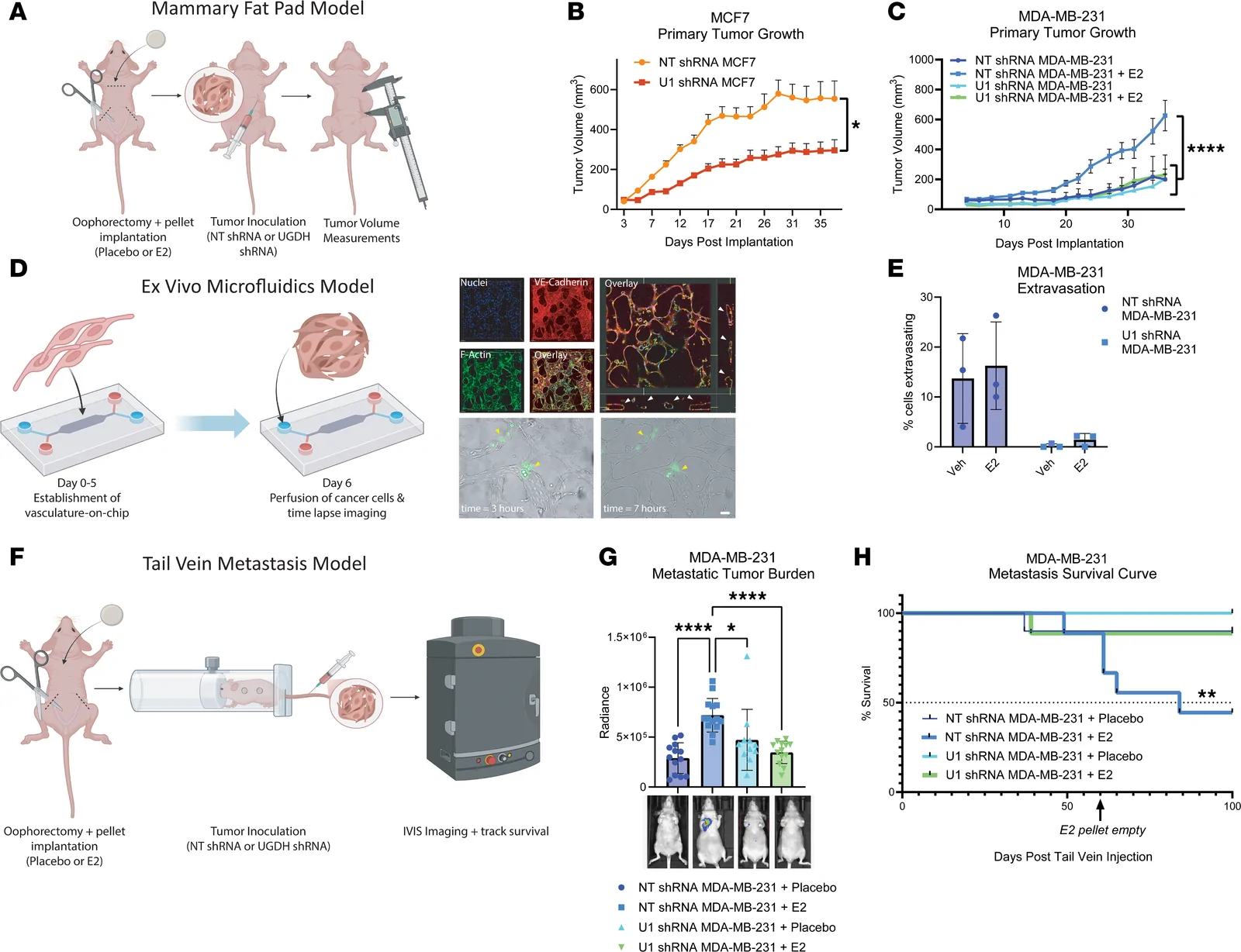
Estrogen promotes tumor growth in breast cancer models in vivo irrespective of ER status, and *UGDH* KD blunts tumor growth and improves animal survival. (**A**) Experimental design of mammary fat pad in vivo model. (**B**) Primary tumor growth curve in mammary fat pad model comparing NT shRNA MCF7 and U1 shRNA MCF7. Student’s *t* test (2-tailed). (**C**) Primary tumor growth curve in mammary fat pad model comparing NT shRNA MDA-MB-231 and U1 shRNA MDA-MB-231. One-way ANOVA. (**D**) Experimental design of breast cancer extravasation model ex vivo depicting generation of endothelium vasculature and time lapse imaging (0 to 7 hours) of GFP-labeled extravasating cells. White scale bar (bottom): 100 µm. Arrowheads represent cells that are extravasating. (**E**) Percentage of NT shRNA and U1 shRNA MDA-MB-231 cells extravasating (*n* = 3 technical replicates). (**F**) Experimental design of tail vein metastasis model using MDA-MB-231 cells. (**G**) Radiance values of imaged mice at week 7 (peak signal) of metastasis experiment with representative bioluminescent images of mice treated with estrogen or placebo pellets and implanted with NT shRNA or U1 shRNA MDA-MB-231 (*n* = 10 mice per group). (**H**) Survival curve of metastasis experiment (*n* = 10 mice per group). Gehan-Breslow-Wilcoxon test; *P < 0.05; **P < 0.01; ****P < 0.0001.

Given the effects of estrogen and *UGDH* KD on breast cancer cells in vitro, we assessed whether *UGDH* KD alters E2-mediated primary tumor growth in vivo. ER^+^ MCF7 cells expressing either an NT control shRNA or *UGDH* KD shRNAs (U1 and U2) were injected into the mammary fat pad of nude mice (Figure 3A). *UGDH* KD significantly decreased the growth rate and reduced the final tumor size of MCF7 tumors by 53% (Figure 3B, *P* < 0.05). Using a similar experimental paradigm in ER^–^ MDA-MB-231 cells, E2-treated NT shRNA tumors showed the greatest growth rate (final tumor volume = 625.9 mm^3^ at day 36, *P* < 0.0001), which was completely abrogated by *UGDH* shRNA (~70% inhibition) (Figure 3C). To evaluate whether E2 stimulation of ER^–^ tumor growth was cell line specific, a primary xenograft breast cancer model using MDA-MB-468 cells with NT shRNA and U1 shRNA, treated with and without E2, was also performed (Supplemental Figure 3A). Again, E2 promoted the greatest primary tumor growth in the NT shRNA MDA-MB-468 line (final tumor volume = 368.4 mm^3^ at day 80, *P* < 0.05), which once again was abrogated by *UGDH* shRNA (44% inhibition).

*UGDH* plays a critical role in the tumor microenvironment, and prior studies demonstrate that it may be implicated in metastasis. Given the estrogen-mediated migration and invasion phenotype in vitro and primary tumor growth response in vivo, we sought to examine the effects of estrogen and *UGDH* KD on the initial steps of breast cancer metastasis. MDA-MB-231 NT shRNA or *UGDH* shRNA cell lines were perfused through an ex vivo microfluidic chip with endothelial vasculature to model human microcirculation and extravasation during the metastatic cascade (Figure 3D). *UGDH* KD significantly reduced the percentage of extravasating MDA-MB-231 cells, regardless of estrogen stimulation (~16-fold; *P* = 0.0284) (Figure 3E). Given that *UGDH* KD blunted the E2-induced primary tumor growth response in vivo and significantly reduced extravasation in the microfluidic ex vivo model, we examined the effects of *UGDH* KD on metastatic tumor progression in vivo. Nu/J mice implanted with either placebo or E2 pellets were injected via tail vein with either control (NT shRNA) or *UGDH* KD MDA-MB-231 cells (U1 shRNA) (Figure 3F). E2-treated control mice showed the greatest metastatic tumor burden compared with placebo-treated control mice (*P* < 0.001); placebo-treated *UGDH* KD mice (*P* = 0.0001); and E2-treated *UGDH* KD mice (*P* = 0.0011) (Figure 3G). All *UGDH* KD-injected mice and placebo-control mice survived significantly longer (median survival undefined, *P* = 0.0101) than E2-treated control mice (median survival = 84 days) (Figure 3H). In a confirmatory survival study in a tail vein metastasis model using MDA-MB-468, E2-treated control mice once again showed significantly worse survival compared with placebo-treated *UGDH* KD mice (*P* < 0.05) and control mice (*P* < 0.05) (Supplemental Figure 3B).

### Estrogen-mediated migratory phenotypes may be potentiated by GPER1 activation.

Estrogen-stimulated migratory and invasion phenotypes, as well as abrogation by *UGDH* KD, were observed in both ER^+^ and ER^–^ breast cancer lines, implying that the effects were not due to direct signaling through the ER. Thus, we hypothesized that the E2 effects may be mediated through the *GPER1/GPR30* (*GPR30*) pathway in both MCF7 and MDA-MB-231 cells. *GPR30* protein expression was assessed in ER^+^ and ER^–^ cell lines in the presence and absence of E2 and a *GPR30* agonist, G1, at various doses to evaluate the optimal dose to activate *GPR30* with G1 (Supplemental Figure 4, A and B). For both the MCF7 and MDA-MB-231 cell lines, there was increased expression of *GPR30* in response to E2 and G1 in the control cell lines, which was not seen in the *UGDH* KD lines. To assess whether the *GPR30* pathway is implicated in resistance to endocrine therapies, we used single-cell sequencing to assess clinical specimens (breast cancer spine metastasis and adjacent normal tissue) obtained from a patient who had received several endocrine-based therapies that had failed. *GPR30* expression was more expressed in the patient who had developed resistance to endocrine therapies than in a patient receiving endocrine therapy (Supplemental Figure 4C).

**Figure 4:**
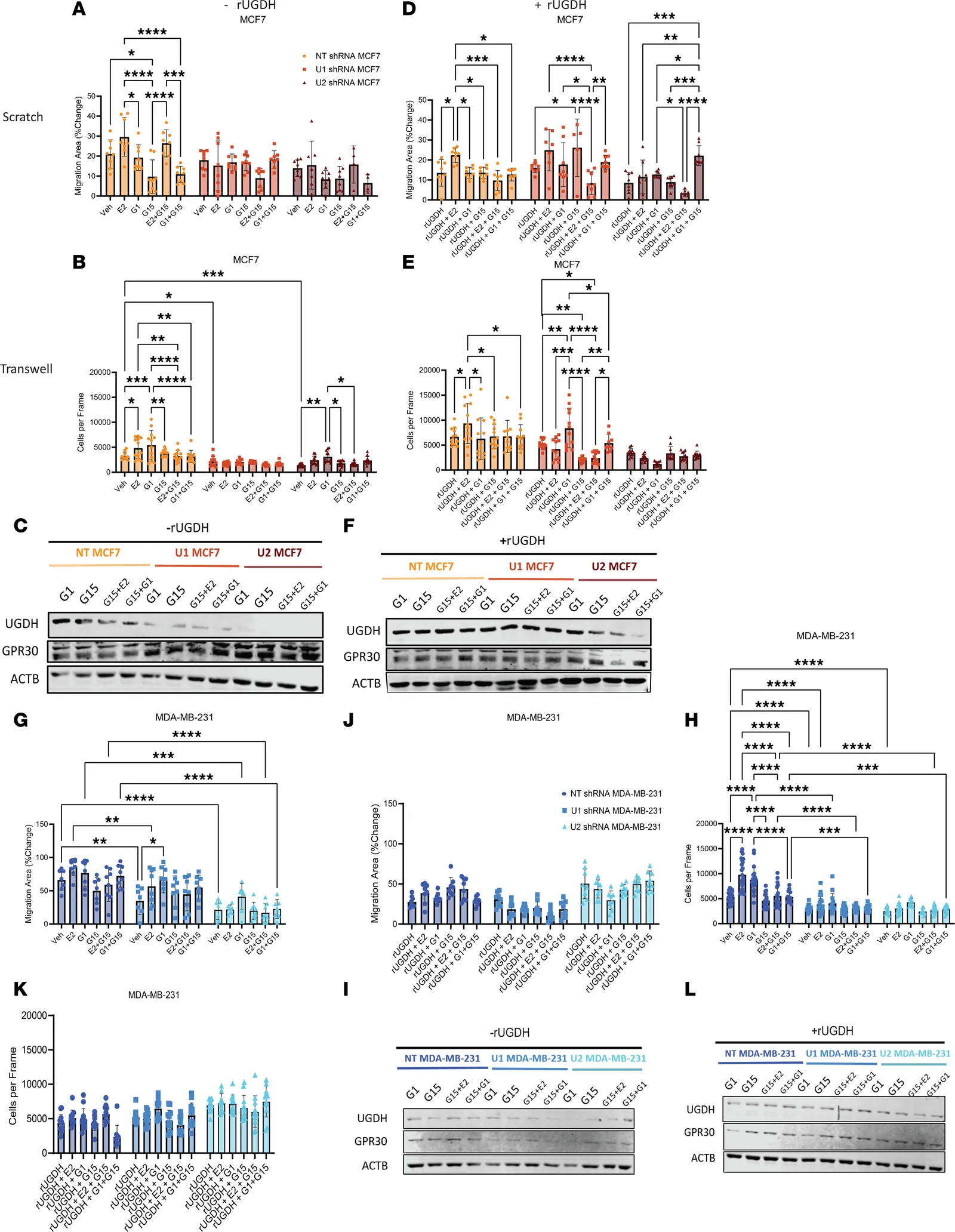
Estrogen-mediated invasion and migration affected by *UGDH* KD may be potentiated by GPR30 activation in ER^–^ breast cancer. (**A**) Scratch and (**B**) Transwell assays in MCF7, comparing effects of E2, G1, G15, E2+G15, and G1+G15 to vehicle treatment in NT shRNA, U1 shRNA, and U2 shRNA, with corresponding (**C**) *UGDH* and *GPR30* protein expression for each treatment condition. (**D**) Scratch and (**E**) Transwell assays in add-back *rUGDH* experiments in MCF7, comparing treatment conditions and shRNAs above with corresponding (**F**) *UGDH* and *GPR30* protein expression for each treatment condition. (**G**) Scratch and (**H**) Transwell assay in MDA-MB-231, comparing effects of E2, G1, G15, E2+G15, and G1+G15 to vehicle treatment in NT shRNA, U1 shRNA, and U2 shRNA, with corresponding (**I**) *UGDH* and *GPR30* protein expression for each experimental condition. (**J**) Scratch and (**K**) Transwell assays in add-back *rUGDH* experiments in MDA-MB-231, comparing treatment conditions and shRNAs above with corresponding (**L**) *UGDH* and *GPR30* protein expression for each treatment condition. One-way ANOVA; *P < 0.05; **P < 0.01; ***P < 0.001; ****P < 0.0001.

To determine whether the *GPR30* pathway alters E2-induced migratory phenotypes, wound healing and Transwell assays comparing E2 and G1 stimulation in combination with a *GPR30* antagonist (G15) were performed in ER^+^ and ER^–^ cell lines with and without *UGDH* shRNA. In the MCF7 line, G1 stimulation did not increase wound healing in control NT shRNA compared with E2 stimulation alone (Figure 4A). Transwell migration was significantly increased with E2 or G1 stimulation alone, and both responses were reversed with G15 treatment (Figure 4B, *P* < 0.01). *UGDH* shRNAs did not show any marked differences among all treatment groups tested. *UGDH* protein expression was decreased with *GPR30* treatments in control NT shRNA, with no effect on *GPR30* expression in control or *UGDH* shRNAs (Figure 4C). Add-back treatment with recombinant *UGDH* (*rUGDH*) restored migration phenotypes in *UGDH* shRNAs to those of NT shRNA in MCF7 in both wound healing and Transwell assays, excluding *UGDH* shRNA2 in the Transwell assay (Figure 4, D–F).

In MDA-MB-231, both E2 and G1 stimulation alone significantly increased wound healing and invasion in NT shRNA compared with *UGDH* shRNAs, which were again reversed with G15 treatment (Figure 4G, *P* < 0.01; Figure 4H, *P* < 0.0001). Unlike in MCF7, *UGDH* shRNAs in MDA-MB-231 showed decreased GPR30 protein expression compared with NT shRNA, suggesting an interaction between *UGDH* and *GPR30* in MDA-MB-231 (Figure 4I). Treatment with *rUGDH* restored migratory phenotypes in *UGDH* shRNAs and similarly *GPR30* expression (Figure 4, J–L).

### RNA-seq of UGDH KD breast cancer lines suggests involvement of growth factor signaling, EMT, and the mTOR pathway.

**Figure 5:**
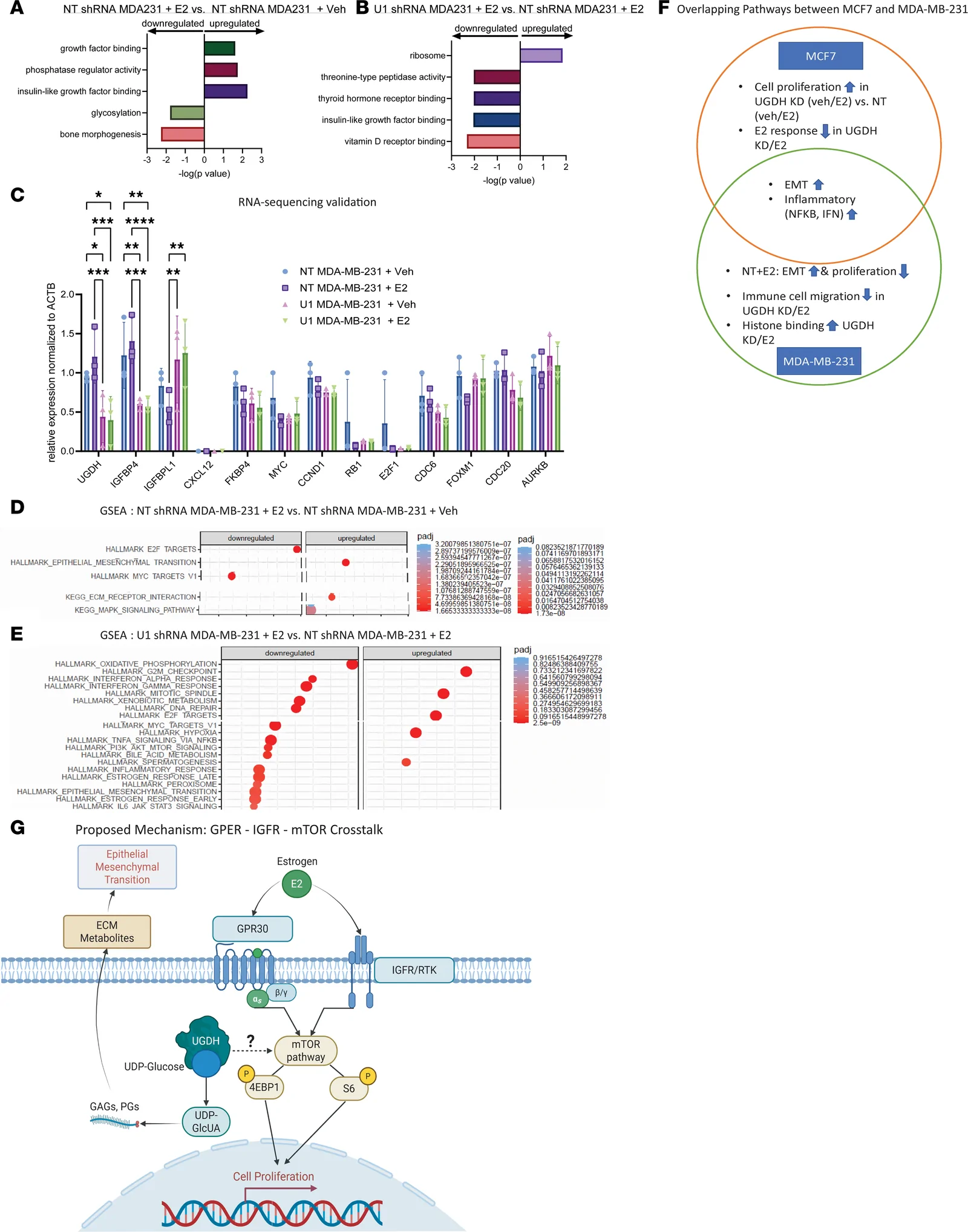
RNA-seq of in vitro samples suggests involvement of growth factor signaling, EMT, and the mTOR pathway. (**A**) Pathway analysis bar graph comparing upregulated and downregulated pathways of NT shRNA MDA-MB-231 + E2 with NT shRNA MDA-MB-231 + vehicle, by –log*P*(value). (**B**) Pathway analysis bar graph comparing upregulated and downregulated pathways of U1 shRNA MDA-MB-231 + E2 with NT shRNA MDA-MB-231 + E2, by –log*P*(value). (**C**) RNA-seq validation of DEGs identified in pathway analysis using qRT-PCR (growth factor and *mTOR* genes). (**D**) GSEA dot plot comparing upregulated and downregulated pathways in NT shRNA MDA-MB-231 + E2 with NT shRNA MDA-MB-231 + vehicle, using Hallmark and KEGG reference domains. (**E**) GSEA dot plot comparing upregulated and downregulated pathways in U1 shRNA MDA-MB-231 + E2 with NT shRNA MDA-MB-231 + E2, using KEGG reference domain. (**F**) Venn diagram summarizing the primary overlapping pathways between MCF7 and MDA-MB-231. (**G**) Proposed mechanism of potential *GPR30*/*IGF1R*/*mTOR* crosstalk; made on BioRender.com. One-way ANOVA; *P < 0.05; **P < 0.01; ***P < 0.001; ****P < 0.0001.

To assess alternative pathways associated with E2-stimulated migratory phenotypes that could be connected to *GPR30*, we performed bulk RNA-seq on NT shRNA cells treated with vehicle or E2 and U1 shRNA cells treated with vehicle or E2 in MCF7 (data not shown) and MDA-MB-231 (Figure 5). Using the bulk sequencing data from MDA-MB-231 and MCF7, pathway analysis of E2-treated control cells compared with vehicle-treated control cells showed upregulation of growth factor binding, phosphatase regulator activity, and IGF binding pathways and downregulation of glycosylation and bone morphogenesis pathways in E2-treated control cells (Figure 5A). Pathway analysis comparing E2-treated *UGDH* KD cells with E2-treated control cells showed upregulation in ribosome-related pathways and downregulation in threonine-type peptidase activity, thyroid hormone receptor binding, IGF binding, and vitamin D receptor binding pathways in E2-treated *UGDH* KD cells (Figure 5B). Given the overlap in IGF binding pathways in both pathway analyses, we validated DEGs that were highlighted in our pathway analysis, including IGF binding proteins *IGFBP4* and *IGFBPL1* and downstream genes related to the *mTOR* pathway, including but not limited to *E2F1*, *MYC*, and *FOXM1*. Both *IGFBP4* and *IGFBPL1* were significantly differentially expressed between control and *UGDH* KD cells, regardless of treatment status (Figure 5C, *P* < 0.01, *P* < 0.001).

Gene set enrichment analysis (GSEA) using Hallmark and KEGG reference libraries confirmed the downregulation of *E2F* and *MYC* pathways and upregulation of EMT and extracellular matrix receptor pathways in E2-treated control cells compared with vehicle-treated control cells (Figure 5D and Supplemental Table 1, *P* < 0.0001). GSEA using the Hallmark reference library confirmed the downregulation of *E2F*, *MYC*, *EMT*, and *mTOR* signaling in E2-treated *UGDH* KD cells compared with E2-treated control cells (Figure 5E and Supplemental Table 2, *P* < 0.0001). Western blot analysis confirmed that *mTOR*-related proteins *CCND1*, *pRB*, *p4EBP1*, *pS6*, and *FOXM1* were downregulated in E2-treated *UGDH* KD (Supplemental Figure 5A). No changes in protein expression of growth factor receptors (such as *EGFR* and *IGF1R*) or the *AKT/ERK* pathways were identified. To assess the extent to which treatment status and *UGDH* status affected the pathway analyses, principal component analysis (PCA) for MCF7 and MDA-MB-231 cells was performed (Supplemental Figure 5B). PCA plots showed that although DEGs and pathways in MCF7 cells were segregated by E2 treatment status, DEGs and pathways in MDA-MB-231 cells did not show any segregation by treatment or *UGDH* status. Overlapping pathways between MCF7 and MDA-MB-231 showed that EMT and inflammatory pathways were the primary shared pathway that was upregulated in E2-treated controls in both cell lines compared with E2-treated *UGDH* KD cells (Figure 5F).

## Discussion

Our study provides further evidence of the role of estrogen in the development of metastatic progression, irrespective of ER status, and establishes the involvement of *UGDH* in estrogen-induced breast cancer progression. Specifically, we demonstrate that estrogen induces migratory and invasive phenotypes in both ER^+^ and ER^–^ breast cancer in vitro and in vivo, which are abrogated by *UGDH* KD. Additionally, *UGDH* KD significantly reduces estrogen-dependent tumor growth in ER^+^ and ER^–^ primary breast cancer orthotopic models in vivo. An ex vivo microfluidics model demonstrated that *UGDH* can regulate extravasation in the presence and absence of estrogen. Also, *UGDH* KD blunted the metastatic formation and progression of ER^–^ breast cancer lines stimulated by estrogen in tail vein models of metastasis in vivo. Furthermore, *UGDH* expression was upregulated in metastatic breast cancer tissue compared with normal vertebral tissue in a patient, specifically in cancer epithelial cells. Our study also identifies the *GPR30* pathway as a potential noncanonical estrogen-mediated signaling pathway that can mediate tumorigenic phenotypes in ER^+^ and ER^–^ breast cancer and is influenced by *UGDH* KD. In the context of in vitro, in vivo, and clinical findings, *UGDH* appears to be involved in breast cancer progression, and mitigating *UGDH* expression can abrogate estrogen-induced migratory phenotypes.

Estrogen is known to increase the risk of breast cancer development (37) via activation of ERα (*ESRA*) in ER^+^ breast cancer, with subsequent upregulation of genes associated with tumor cell invasion, growth, and survival (38). Although ER^–^ breast cancer is not widely considered estrogen responsive, previous literature (39) suggests that ER^–^ cell lines can become more aggressive in the presence of estrogen, which is consistent with our findings. From a therapeutic context, ER degraders may result in downregulation of canonical pathways; however, the estrogen ligand would still be available to stimulate noncanonical pathways even in the presence of endocrine therapy resistance. Mechanistically, crosstalk between estrogen stimulation, growth factor signaling pathways involving receptor tyrosine kinases such as *IGF1R* and *EGFR*, and cell surface receptors including G protein–coupled receptors (GPCRs) and their downstream mechanisms have been implicated in estrogen effects that are ER independent (13, 39–45). Activation of *GPR30* by estrogen, anti-estrogen compounds (immune checkpoint inhibitor or tamoxifen), and *GPR30* selective agonists (G1) are shown to increase migratory and/or proliferative breast cancer phenotypes, irrespective of ER status, and there is an increasing body of literature to suggest that its expression is associated with poor outcomes in breast cancer, which is concordant with our findings (Supplemental Figure 4C). *IGF1R* signaling is also involved in EMT-mediated metastatic tumor progression, and *IGF1R* overexpression is shown to promote migratory phenotypes of triple-negative breast cancers via the focal adhesion kinase signaling pathway (32, 33). Additionally, recent work has linked increased *GPR30* expression to both worse survival and pro-metastatic phenotypes in patients with ER^–^ breast cancer and postmenopausal patients with ER/PR^+^ breast cancer (13, 45).

Congruent with our findings, previous studies also demonstrated that *UGDH* KD abrogates tumor growth, invasion, and colony formation but does not affect proliferation in triple-negative breast cancer cell lines (21, 46), glioblastoma (23), and lung adenocarcinoma (24). Conversely, *UGDH* KD can mitigate both tumor cell proliferation and migration (47) in colorectal carcinoma and in ovarian cancer (22). Although our findings align with the previously proposed “go-or-grow” hypothesis that cancer cell motility and proliferation can be mutually exclusive (48, 49), it is important to note that this dichotomy is not ubiquitous across all cancer types. Dysregulation in the *UGDH*-driven glycosaminoglycan/proteoglycan production pathway has been described as a possible explanation for impairment of cancer cell motility by *UGDH* KD (14). Taking our results in the context of recent glioblastoma studies, *UGDH* may aid in generating extracellular matrix precursors that influence cancer cell migration, EMT processes, and macrophage-related transcriptional and epigenetic regulatory pathways to modulate estrogen-induced tumorigenic phenotypes (38).

To our knowledge, this is the first study demonstrating that *UGDH* expression can mediate the tumorigenic effects of hormone stimulation via estrogen in breast cancer regardless of ER status. Of the noted ER-independent pathways, we show that *UGDH* KD can alter the *GPR30* pathway and associated estrogen-induced and *GPR30* activation–induced tumor phenotypes (19, 33, 34). Although *GPR30* has been shown to play a role in ER^+^ and ER^–^ breast cancer, no previous work to our knowledge has linked *UGDH* to this receptor (20). Additionally, although we demonstrate correlation between *UGDH* and *GPR30* expression and association of the *mTOR* pathway and EMT mechanisms, secondary messenger signaling pathways associated with *UGDH/GPR30* interaction are unclear and have yet to be explored. A potential explanation could be that *UGDH* interacts with signaling downstream of *GPR30* to activate and/or stabilize EMT-related pathways in breast cancer, like in lung cancer (24). This mechanistic hypothesis is supported by our data demonstrating downregulation of EMT signaling in E2-treated *UGDH* shRNA cells compared with E2-treated control cells, which was validated by qPCR (Supplemental Figure 6). In line with this hypothesis, our bulk RNA-seq data showing downregulation of IGF binding pathways in both ER^+^ and ER^–^ cell lines with *UGDH* shRNA provides additional insight into a possible mechanistic interaction between *UGDH*, *GPCR*, and EMT activation, as *IGF1R* signaling has been shown to mediate EMT-driven metastasis and migration in breast cancer (20, 50, 51).

The *mTOR* pathway, which is downstream of both *IGF1R* and *GPR30*, has multiple documented mutations in breast cancer that can affect cell growth, migration, proliferation, apoptosis, metabolism, and DNA repair, and patients with breast cancer are routinely tested for genetic variations in this pathway. Although no literature specifically links *UGDH* or *GPR30* to the *PI3K/AKT/mTOR* pathway in breast cancer, several studies report interactions between *UGDH*, growth factor receptors, and this pathway that result in increased cancer cell motility, invasion, migration, and survival (52, 53). Additionally, our RNA bulk sequencing data suggest that interactions between *UGDH* and the *mTOR* pathway may be critical in both ER^+^ and ER^–^ breast cancers given that IGF binding proteins and downstream genes in the *mTOR* signaling pathway were differentially expressed in *UGDH* shRNA versus controls. Our analyses highlight the potential importance of this pathway regardless of ER status and estrogen stimulation. Taken together, we propose a mechanism in which *UGDH* is modulating the production of extracellular matrix metabolites to stabilize the cell membrane for E2-mediated *GPR30* or *IGF1R* activation, which may activate *mTOR* signaling downstream to stimulate migration and proliferation. Although our model incorporates speculative components from our RNA-seq that require further validation in future experiments, we suspect that, in the presence of estrogen, *UGDH* may induce a bidirectional crosstalk between *GPR30* and IGFR/RTKs to upregulate the *mTOR* pathway and resulting cell proliferation. Concurrently, by stimulating EMT through *UGDH* and downstream pathways, *UGDH* can promote breast cancer cell migration (Figure 5G). These findings provide a potential alternative intrinsic signaling pathway that may contribute to endocrine therapy resistance and could be targeted in patients with breast cancer irrespective of ER status. Furthermore, we have identified 2 potential therapeutic targets (*UGDH* and *GPR30*) to combat estrogen-mediated tumorigenesis across multiple breast cancer subtypes.

Our study provides further evidence for the role of estrogen in the development of metastatic progression, irrespective of ER status, and establishes the involvement of *UGDH* in estrogen-induced breast cancer progression. *UGDH* KD effectively blunted estrogen-stimulated phenotypes in breast cancer in vitro and in vivo irrespective of ER status. Additionally, we identify a noncanonical, non-ER signaling pathway in breast cancer that can be targeted through a *UGDH/GPR30* axis, irrespective of ER status. These studies support the role of estrogen and *UGDH* in breast cancer progression and provide a foundation for future studies to evaluate the role of *UGDH* and *GPR30* in therapeutic resistance to improve outcomes and survival for patients with breast cancer.

## Methods

### Sex as a biological variable

Our study exclusively examined female mice because the disease modeled (breast cancer) is predominantly relevant in females and rarely affects males.

### Reagents and cell culture

All reagents were purchased from Sigma-Aldrich unless otherwise specified. Puromycin was diluted to a concentration of 1 μg/mL in cell culture medium as a working concentration. MCF7:WS8, T47D, and MDA-MB-231 cell lines (American Type Culture Collection, ATCC) were grown in DMEM (11965092, Thermo Fisher Scientific), RPMI 1640 (11875093, Thermo Fisher Scientific), and MEM (11095080, Thermo Fisher Scientific) respectively, all supplemented with 10% FBS (A5256701, Thermo Fisher Scientific), nonessential amino acids (1%) (11140050, Thermo Fisher Scientific), sodium pyruvate (1%) (11360070, Thermo Fisher Scientific), and puromycin (A1113802, Thermo Fisher Scientific). Versions of the above media without phenol red were used for in vitro experiments investigating the effects of hormone stimulation to remove endogenous hormone use in phenol red (DMEM: 21063029; RPMI: 11835030; MEM: 51200038, Thermo Fisher Scientific). E2 (E-060, Sigma-Aldrich) was used in all assays at a physiological concentration of 1 nM/mL. For *GPR30* treatment-related experiments, *GPR30* agonist G1 (3577, Tocris Bioscience) was used at a final concentration of 0.1 μm and *GPR30* antagonist G15 (3678, Tocris Bioscience) was used at a final concentration of 1 μm. For *rUGDH* add-back experiments, human recombinant *UGDH* from *E*. *coli* (ENZ-535, PROSPEC Protein Specialists) was used at a final concentration of 6 μm. To assess the effect of hormonal stimulation, *GPR30* stimulation, and add-back of *UGDH*, twice charcoal-stripped (CS) media at either 10% or 0.5% (A3382101, Thermo Fisher Scientific) was used for all assays.

### Lentiviral transduction

*UGDH* shRNA lentiviral particles were purchased from Dharmacon as a set of 3 SMARTvector Inducible Human *UGDH* shRNA (V3SH7675-01EG7358). Control (NT) shRNA clone (VSC6570), *UGDH* shRNA1 (V2LHS_171838), and *UGDH* shRNA3 (V2LHS_218865) were used. MCF7, T47D, and MDA-MB-231 cells were transduced with virus in polybrene media (8 μg/mL) (TR-1003, 50 μL, Sigma-Aldrich) for 48 hours prior to puromycin selection (1 μg/mL).

### Quantitative real-time PCR

Total RNA was extracted via Aurum Total RNA Mini kit (7326820, Bio-Rad). After reverse transcription using iScript cDNA Synthesis kit (1708891, Bio-Rad) and Oligo(dT) primer, quantitative real-time PCR (qRT-PCR) was performed using iScript SYBR Green-based Reverse Transcription Supermix (1708841, Bio-Rad). Relative gene expression was normalized to *36B4* or *ACTB* gene expression.

Primers used for qRT-PCR experiments were as follows: *UGDH* (forward: GAGGTAGCAACAGCAACAGCGATTGG; reverse: TCTGGCAAATTCAGAGCCTCA); *36B4* (forward: GGACATGTTGCTGGCCAATAA; reverse: GGGCCCGAGACCAGTGTT). Primers used for the Supplemental figure experiments were as follows: *ESR1* (forward: CCCTCCACACCAAAGCATCTG; reverse: GCGGCGTTGAACTCGTAGG); *SNAI1* (forward: CGAGTGGTTCTTCTGCGCTA; reverse: GGGCTGCTGGAAGGTAAACT); *CDH1* (forward: ATTCTGATTCTGCTGCTCTTG; reverse: AGTAGTCATAGTCCTGGTCTT); *CDH2* (forward: GATCTGGGTCCTGAGCAGTG; reverse: TCAGTCAACTGAACCGTGT); *IGF1R* (forward: GCTGGTGGATGCTCTTCAGT; reverse: ACTCATCCACGATGCCTGTC); *CCND1* (forward: TGTCCTACTACCGCCTCACA; reverse: CTTGGGGTCCATGTTCTGCT); *RB1* (forward: TTGGATCACAGCGATACAAACTT; reverse: AGCGCACGCCAATAAAGACAT); *E2F1* (forward: ACG TGA CGT GTC AGG ACC T; reverse: GAT CGG GCC TTG TTT GCT CT); *FOXM1* (forward: GCGGAGCGTTAAGGTCAC; reverse: CCACGGCCACTTCTTCCC); *CXCL12* (forward: AGCCAACGTCAAGCATCTCA; reverse: TCGGGTCAATGCACACTTGT); *IGFBP4* (forward: GGGGATGCCCTGCGGGGTGTA; reverse: CGAAGTGCTTCTGCAGGCACCT); *IGFBPL1* (forward: TATAGCTGTCCAAGTGCGAGGG; reverse: ATGTTGGCTGCATGGCACTGGT); *MYC* (forward: CATGAGGAGACACCGCCC; reverse: GCCTGCCTCTTTTCCACAGA); *AURKB* (forward: ATCAGCTGCGCAGAGAGATC; reverse: AGCTCTTCTGCAGCTCCTTG); *FKBP4* (forward: CGTGGAGGTTGCACTGGAAGGG; reverse: TCCATGCGCTGAATGGCCCTC); *CDC20* (forward: TCGCATCTGGAATGTGTGCT; reverse: GGTTCTGTGCAAAGCCATGG); *CDC6* (forward: GCAGTTCAATTCTGTGCCCG; reverse: TAGCTCTCCTGCAAACATCCAG).

### Immunoblot and immunocytochemistry

Total cellular protein was extracted with RIPA buffer containing protease inhibitors (78425, Thermo Fisher Scientific) and sodium orthovanadate (J601919.AD, Thermo Fisher Scientific). SDS-PAGE was performed with 50 μg total proteins prepared using premade sample loading buffer reagents (1610747, Bio-Rad) using 10% gradient acrylamide gels (TEMED: 1610801; ammonium persulfate: 1610700; 30% acrylamide/bis solution, 29:1, 161046, Bio-Rad). Western blot imaging was performed using an Odyssey CLx imaging system (LI-COR Biosciences). Western blot analysis was performed using a quantitative Western blot system, with secondary antibodies labeled by the following IRDye infrared dyes: goat mouse IgG (680/701 nm) (89138-516, Biotium) and goat-rabbit IgG (770 nm) (89138-536, Biotium). Antibodies included in primary experiments were the following: anti-*UGDH* (ab15505, Abcam), anti-*ESR1*, anti-*GPR30*, and anti-*ACTB* (8H10D10, Cell Signaling Technology). Other antibodies used in the Supplemental Data were the following: anti-*EGFR* (4267, Cell Signaling Technology), anti-*IGFRB* (9750, Cell Signaling Technology), anti-*HER2/ERbB2* (4290, Cell Signaling Technology), anti-*4EBP1* (9452, Cell Signaling Technology), anti-*AKT* (9272, Cell Signaling Technology), anti-*CCND1* (55506, Cell Signaling Technology), anti-*E2F1* (3742, Cell Signaling Technology), anti-*ERK* (4695, Cell Signaling Technology), anti*-FOXM1* (20459, Cell Signaling Technology), anti-*C-MYC* (13987, Cell Signaling Technology), anti-phospho*-4EBP1* (2855, Cell Signaling Technology), anti-phospho*-AKT* (9271S, Cell Signaling Technology), anti-phospho*-ERK* (MAB148, Invitrogen), anti-phospho*-IGFRB* (9750, Cell Signaling Technology), anti-phospho*-RB* (8516, Cell Signaling Technology), anti-phospho*-S6* (4858T, Cell Signaling Technology), anti-*RB1* (9313, Cell Signaling Technology), and anti-*S6* (2217T, Cell Signaling Technology).

### Proliferation assay

First, 2,000 cells per well were plated in a 96-well plate and starved in 10% CS FBS-supplemented media for 48 hours and then stimulated with either 1 nM E2 or continued 10% CS media. Cell viability assays were performed using CellTiter-Glo (G9241, Promega); reading was taken at baseline (pretreatment) and daily until day 6.

### Wound healing assay

Cells were grown in 10% CS serum in 6-well plates until confluent. Three scratches per well were created using a wide-tip 10 μL pipette tip through the confluent cells. Dishes were washed with PBS and cells were grown in 0.5% CS medium with or without estrogen stimulation for 24–48 hours. Phase contrast pictures were taken at 0, 24, and 48 hours. The width of the scratch was measured and quantified using the MRI Wound Healing plugin in ImageJ (NIH).

### Transwell migration assay

Cells were grown in 10% CS serum in 6-well plates for 24 hours; they were then either starved with 0.5% CS (control) or treated with 1 nM E2 for 24 hours. Transwell inserts (3422, Corning) were placed into 24-well plates and seeded at 50,000–75,000 cells per insert in 100 L of either 0.5% CS media or 0.5% CS media supplemented with or without E2 for 24 hours. Cells migrated toward media with 10% CFS for 12–24 hours and then were fixed with 30% formaldehyde, stained with crystal violet (212525, BD Diagnostic Systems) and then washed with PBS. Pictures of the entire Transwell surface were taken, and cell counts were quantified using ImageJ.

### Ex vivo microfluidic metastasis model

The microfluidic vasculature-on-a-chip device was used to assess cancer cell extravasation ex vivo. The device was fabricated using soft lithography as reported (54, 55). Briefly, SU-8 100 photoresist (SU-8-50-100, Microchem, Kayaku Advanced Materials) was spun on a 4-inch silicon wafer (201972, University Wafers) and cured via light exposure through a photomask to create features with a height of 100 μm. The uncured SU-8 was washed away using SU-8 developer, and the wafer was silanized using Trichloro(1H,1H,2H,2H-perfluorooctyl)silane (448931, Sigma-Aldrich). Sylgard 184 (4019862, Ellsworth Adhesives) polydimethylsiloxane (PDMS) at 10:1 (base/crosslinker) was poured onto the wafer and allowed to cure at 60**°**C for 2 hours. After curing, the PDMS was cut around the features; then, inlets and outlets were punched using biopsy punches and bonded to a cover glass using plasma treatment for 60 seconds. The device consisted of 3 parallel microchannels with an array of trapezoidal micro-posts separating the 3 channels. HUVECs (C2519A, Lonza Bioscience) were suspended in 4 U/mL thrombin solution at a concentration of 50 million/mL, mixed 1:1 with 5 mg/mL fibrinogen solution, and perfused into the central microchannel of the device to allow gelation. After gelation, media channels were flushed with cell culture medium and the devices were cultured for 5 days to allow the endothelial cells to self-assemble into microvascular networks with perfusable lumens. The engineered tissue was immunostained for VE-cadherin (AF938, Bio-Techne) (1:100 dilution) followed by donkey anti-goat (1:200) secondary antibody to visualize the endothelial cell-cell junctions. Alexa Fluor 488 phalloidin (A12379, Thermo Fisher Scientific) and Hoechst 33342 (H3570, Thermo Fisher Scientific) were used to stain actin and nuclei, respectively. Fluorescently labeled control (NT shRNA) and *UGDH* KD (*UGDH* KD1) MDA-MB-231 cell lines were suspended in their growth medium and perfused into the vascular networks in the presence or absence of estrogen (1 nM), and the microfluidic devices were cultured for up to 8 hours to allow the cells to extravasate from the vasculature into the fibrin hydrogel. The number of extravasating cells was assessed per condition using ImageJ software.

### Animal studies

#### Mammary fat pad models.

Mice used in all experiments were female Nu/J (002019, The Jackson Laboratory) aged 6–8 weeks. Sample size was determined by endpoint statistics (power analysis) performed prior to study initiation by James Herndon (Duke University Medical Center, Durham, North Carolina, USA) (*n* = 30). For MCF7 experiments, all mice were ovariectomized and implanted with E2 pellets to promote tumor growth and progression (0.36 mg, 60 days, SE-121, Innovative Research of America). Mice then received mammary fat pad injections of 1.3 × 10^6^ viable MCF7 cells that were stably transfected with the NT shRNA, *UGDH* shRNA1, or *UGDH* shRNA2. Tumor volumes were measured 3 times weekly for 5 weeks. Tumor volumes were calculated using the formula (short width × short width × long width) / 2. After mice were euthanized, primary tumors were removed and processed for RNA and protein analysis. The same protocol was used for the mammary fat pad experiments with MDA-MB-231 and MDA-MB-468 with the following modifications. Mice (*n* = 40) were ovariectomized and implanted with either placebo pellets (SC-111, Innovative Research of America) (groups 1 and 3) or E2 pellets (groups 2 and 4). Groups 1 and 2 were then injected with NT shRNA MDA-MB-231 cells into the mammary fat pad, and groups 3 and 4 received *UGDH* shRNA1 MDA-MB-231 cells. The resulting experimental groups were the following (*n* = 10 mice/group): (a) NT shRNA + placebo; (b) NT shRNA + estrogen; (c) *UGDH* shRNA1 + placebo; (d) *UGDH* shRNA1 + estrogen. Tumor volume measurement and tissue harvest were the same as described above.

#### Metastasis models.

To establish the metastatic breast cancer models, mice (*n* = 60) were ovariectomized and implanted with either E2 pellets or placebo pellets. Parental cells used to generate the luciferase-labeled and shRNA lines below were MDA-MB-231 and MDA-MB-468 (ATCC, Duke University). Mice then received arterial tail vein injections of 1 × 10^6^ viable cells expressing luciferase and infrared protein that were stably transfected with NT shRNA or *UGDH* shRNA1. The resulting experimental groups were the following (*n* = 15 mice/group): (a) placebo pellet + NT shRNA MDA-MB-231 or MDA-MB-468; (b) estrogen pellet + NT shRNA MDA-MB-231 or MDA-MB-468; (c) placebo pellet + *UGDH* shRNA1 MDA-MB-231 or MDA-MB-468; (d) estrogen pellet + *UGDH* shRNA1 MDA-MB-231 or MDA-MB-468.

To track metastatic tumor progression, mice were imaged weekly using the IVIS Lumina III In Vivo Imaging System (PerkinElmer) to quantify tumor burden as normalized radiance. Prior to imaging, mice were first i.p. injected with 100 μL of 15 mg/mL D-luciferin sodium salt (1-360243-200, Regis Technologies) reconstituted in sterile 1× Dulbecco’s PBS. Images were collected at exposure times of 1 second, 30 seconds, 60 seconds, and 180 seconds and quantified as normalized radiance.

Mice were weighed and monitored 3 times weekly for survival until the end of the study. Mice were euthanized when body weight fell below 15% of their original day 0 weights or when mice could no longer ambulate for food or water due to tumor-related paralysis. Upon median survival of any group, *n* = 5 mice per group were euthanized and lung, liver, and uterus were harvested to be weighed and noted for tumor burden and estrogen effects. Mice harvested for the median survival time point or found dead due to nontumor-related causes before week 1 of the study were censored from the final survival curve.

### Cell line sequencing

Gene expression analysis from cell lines was generated by mRNA-seq using Illumina NovaSeq 6000. Briefly, 1 μg of total RNA was converted to RNA-seq libraries using the Kapa Stranded mRNA-seq library prep kit (KK8421, Roche Sequencing) and sequencing on an Illumina NovaSeq 6000 using the 50 bp paired-end configuration with an average of 51.2 million read pairs per sample. Reads were aligned to GRCh38 using STAR (56). Transcript abundance estimates were performed for each sample using Salmon (57). Differential expression analysis was performed using DESeq2 (58) with an FDR cutoff of 5% (i.e., a *P* value of less than 0.05 was considered significant).

### Single-cell sequencing of patient specimens

#### Patient history.

All patient samples for this study are approved under IRB protocol (Pro 00101198). The first patient was a 71-year-old woman with a history of *ER^+^/PR^+^/HER2*^–^ metastatic breast cancer after T10-L2 radiation to the spine of 30 Gy in 10 fractions completed 3 months prior to the surgical procedure. The patient started on palbociclib 1 month later (held for 2 weeks after initiation for a root canal/tooth abscess and then reinitiated). The patient received palbociclib for 2 months until PET scan and MRI findings identified progression of disease at T11 and L1 corresponding to a PET scan (Figure 1, A–C). The patient received biopsy/radiofrequency ablation and kyphoplasty with cement-augmentation. The second patient was a 42-year-old woman with a history of *ER^+^/PR^+^/HER2*^–^ breast cancer initially started on tamoxifen and then switched to palbociclib and letrozole and ovarian function suppression (OFS) 7 months later. The palbociclib was discontinued, and the patient was continued on letrozole/OFS with overall stable disease. She underwent a left mastectomy and lymph node dissection 3 months later, with 5 out of 15 positive lymph nodes and resection of a 6.4 cm primary invasive lobular tumor with neuroendocrine features. She was switched to exemestane 1 year later and then to anastrozole 1 month later because of joint achiness. The patient presented 1 month later with pain in her neck, shoulders, and mid- and lower back, with imaging demonstrating progression of disease. MRI of the total spine with and without contrast demonstrated new and progressive osseous metastatic lesions in the C6 vertebral body and throughout the vertebral column, with epidural spread at the dorsal aspect at C5-6 and the posterior elements. The patient underwent a C5-7 laminectomy and C4-T2 fusion with resection of tumor.

#### Surgical procedure for specimen acquisition.

After the patient was deemed a surgical candidate based on clinical indications and consent obtained for the collection of tissue, the patient was placed under general anesthesia, and the vertebral body (T11-L1) was identified with fluoroscopic guidance. The skin overlying the target area was prepped with chlorhexidine and sterilely draped in the usual fashion, maintaining meticulous sterile technique. Local anesthesia was provided with 1% lidocaine. A stab incision was made 1 cm lateral to the lateral border of each pedicle at the level of the target vertebral body. A trocar was introduced safely through each pedicle just inside the target vertebral body. The stylet of the trocar was removed and a working cannula was left in place. The biopsy cannula was inserted, and anterior/posterior and lateral fluoroscopic x-ray views were taken to verify accurate placement of biopsy cannula (Figure 1D). A biopsy was taken from the representative levels and divided in half. One half of the specimen was sent to pathology for further evaluation to confirm the presence of normal or tumor specimen (Figure 1, E and F) and evaluated for its tissue of origin (Figure 1, G–I). The other half was placed in a pathology specimen cup and taken to the laboratory for further single-cell sequencing processing within 30 minutes of acquisition, preferably transferred on ice to maintain cell viability. The remainder of the procedure was performed according to clinical indications and then closed in the standard fashion.

#### Sample processing for single-cell studies.

Human samples were processed as previously described (59). Briefly, tissues from tumor and normal vertebral bodies were manually dissected from the block of tissue using razor blades, surgical scissors, and forceps to obtain pieces of tissues that were less than 2 grams total. Samples were then minced using a razor blade and placed into a gentleMACS C tube (130-093-237, Miltenyi Biotec) containing serum-free DMEM, 1 mg/mL collagenase A (10103586001, MilliporeSigma), and 0.1 mg/mL DNAse I (D5025-150KU, Sigma-Aldrich). Samples were homogenized on a gentleMACS Dissociator (130-093-235, Miltenyi Biotec) with 2 rounds of the “h_impTumor_02.01” program and then placed into a shaking incubator for 30 minutes at 37°C. Samples were then processed again on the gentleMACS Dissociator with 2 rounds of the “h_impTumor_03.01” program and gently triturated to ensure the tissues were homogenized. Samples were filtered through a 40 μm filter and rinsed with additional sterile-filtered serum-free DMEM. Samples are then centrifuged (10,000*g*, 5 minutes) to pellet the cells and discard the supernatant, then resuspended in ACK lysis buffer and incubated at room temperature for 3–5 minutes. Samples were then diluted with 1× Dulbecco’s PBS (1:10), mixed by inverting, and then centrifuged again (10,000*g*, 5 minutes, at 4°C). Finally, cells were resuspended in Dulbecco’s PBS, counted, and then centrifuged for a final time before being resuspended in cryopreservation media at 10–20 million live cells per milliliter. Cells were frozen slowly at –80°C in cryofreezing containers until ready for further downstream processing in a batch with other samples. Samples were prepared for single-cell RNA-seq using standard RNA isolation, cDNA synthesis, and sequencing library preparation protocols according to 10X Genomics protocols and were processed for single-cell sequencing through the Duke Sequencing and Genomic Technologies research core facility.

#### Sequencing analysis.

Subsequent single-cell RNA-seq data were analyzed using standard Seurat pipeline to perform quality control preprocessing, normalization, identification of highly variable features, scaling, linear dimensionality reduction, and clustering. Cell type identification and annotation were performed using InferCNV analysis (to identify cells with high copy number variations or CNVs, which are likely cancer cells) and SingleR. Uniform manifold approximation and projection (UMAP) plots and heatmaps were generated using R studio.

### Clinical PFS analysis

The association between *UGDH* expression level and PFS in patients with ER^+^ breast cancer was assessed via an online Kaplan-Meier plotter (60). This platform consolidates patient data from The Cancer Genome Atlas project, Gene Expression Omnibus, and Therapeutic Goods Administration and allows for the comparison of PFS for patients with low and high expression of specific proteins and RNA.

### Statistics

In vitro and in vivo experiments were primarily analyzed with Student’s *t* test (2-tailed), 1-way ANOVA, or 2-way ANOVA (if time was an additional variable of analysis) with Bonferroni’s post hoc correction. Survival curves were analyzed with the Gehan-Breslow-Wilcoxon test. A *P* value less than 0.05 was considered significant.

### Study approval

All protocols involving animals were previously approved by the Duke University IACUC. All patient samples for this study are approved under Duke University IRB protocol (Pro 00101198). Written informed consent was received prior to patient participation.

### Data availability

Sequencing data for the in vitro sequencing experiment and human single-cell sequencing data have been deposited in a MINSEQE-compliant public database (NCBI Gene Expression Omnibus number 25469923); further details regarding the human samples are available upon request at the discretion of the corresponding author. The Supporting Data Values file is available as a single Excel file with separate tabs for each applicable figure panel, labeled as “Fig (number) (letter for subpanel).”

## Author contributions

MJP contributed to project conception and design; extensive drafting and revision of the manuscript and figures; design of experiments; and data acquisition, analysis, and interpretation for all experiments in Figures 1–5. ADN contributed to drafting and revising the manuscript and figures; designing experiments; and conducting data acquisition, analysis, and interpretation in Figures 1–5 and generating Supplemental Figures 1–6 and Supplemental Tables 1–4. CHS contributed to data acquisition and analysis for Figures 1 and 5 (RNA-seq of patient data and cell line data) and to data interpretation of Figures 1–3. TT contributed to experimental design and data interpretation of Figure 4, significant manuscript revisions, and the design and reformatting of Figures 1–5 and Supplemental Figures 1–6. CCDB contributed to data interpretation of Figures 2 and 3. CL contributed to data acquisition for Figure 2 (proliferation assays in Figure 2, F and L). JKB contributed to data interpretation of Figures 2 and 3. DM contributed to data acquisition and interpretation of migration assays in Figure 2. SEW and SA contributed to the experimental design, execution, and data interpretation of all animal experiments in Figure 3. VK contributed to the experimental and technological design, data acquisition, analysis, and interpretation of the ex vivo microfluidics model in Figure 3D. SV contributed to the experimental and technological design of the ex vivo microfluidics model in Figure 3D. CRG contributed to drafting and revising the manuscript, project conception and design, and data analysis and interpretation for all figures and experiments.

## Conflict of interest

CRG is a consultant for Medtronic and Icotec and received research grants from Duke Bass Connections, Stryker, and Carlsmed.

## Funding support

This work is the result of NIH funding, in whole or in part, and is subject to the NIH Public Access Policy. Through acceptance of this federal funding, the NIH has been given a right to make the work publicly available in PubMed Central.

- Grants from the Robert Wood Johnson Harold Amos Medical Faculty Development Program and the FDA to CRG.
- NIH/NINDS K12 NRCDP Physician Scientist Award to CRG.

## Supplementary Material

Unedited blot and gel images

Supporting data values

## Version 1 09/08/2026

Electronic publication
