# Supplementary material for: Estrogen promotes tumor phenotypes in ER+ and ER– breast cancer through UGDH-GPR30
Source: JCI Insight. 2026 Sep 8;11(17):e197357. doi: 10.1172/jci.insight.197357 (PMC13564090; doi:10.1172/jci.insight.197357)
Supplement: Supplementary file 1 [file jciinsight-11-197357-s010.pdf]

Figure 2B: MCF7 Protein Expression of UGDH

Home

	NT shRNA MCF7	U1 shRNA MCF7	U2 shRNA MCF7
Veh	11.94130926	2.615694165	4.119922631
E2	15.69518717	2.619392185	4.258064516

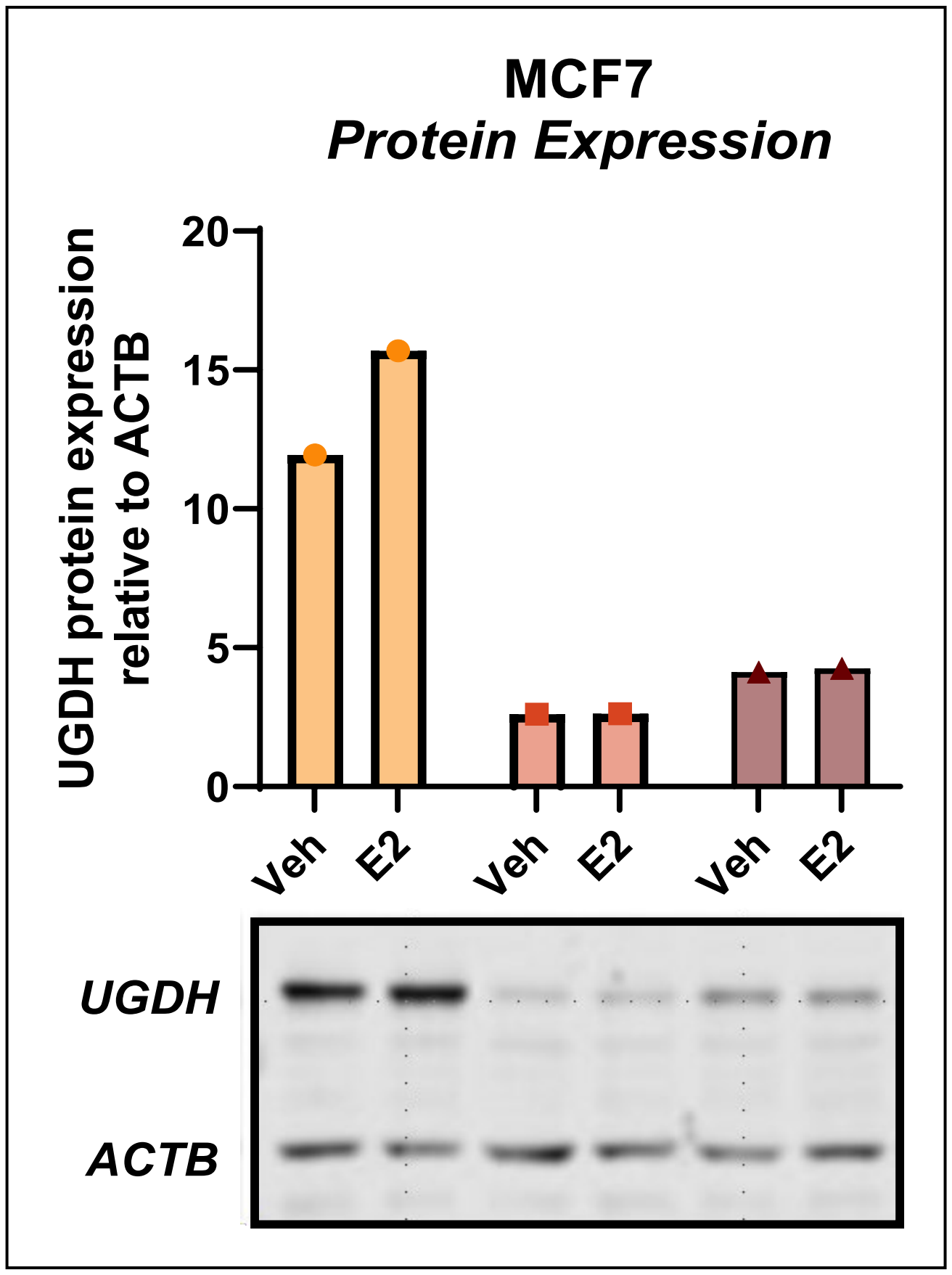

Re-run of experiment for Figure 2B. The original gel above was cut and the entire gel is represented in the image above. We have included an alternative version of Figure 2 with the below gel in place in case this would meet the journals standards.

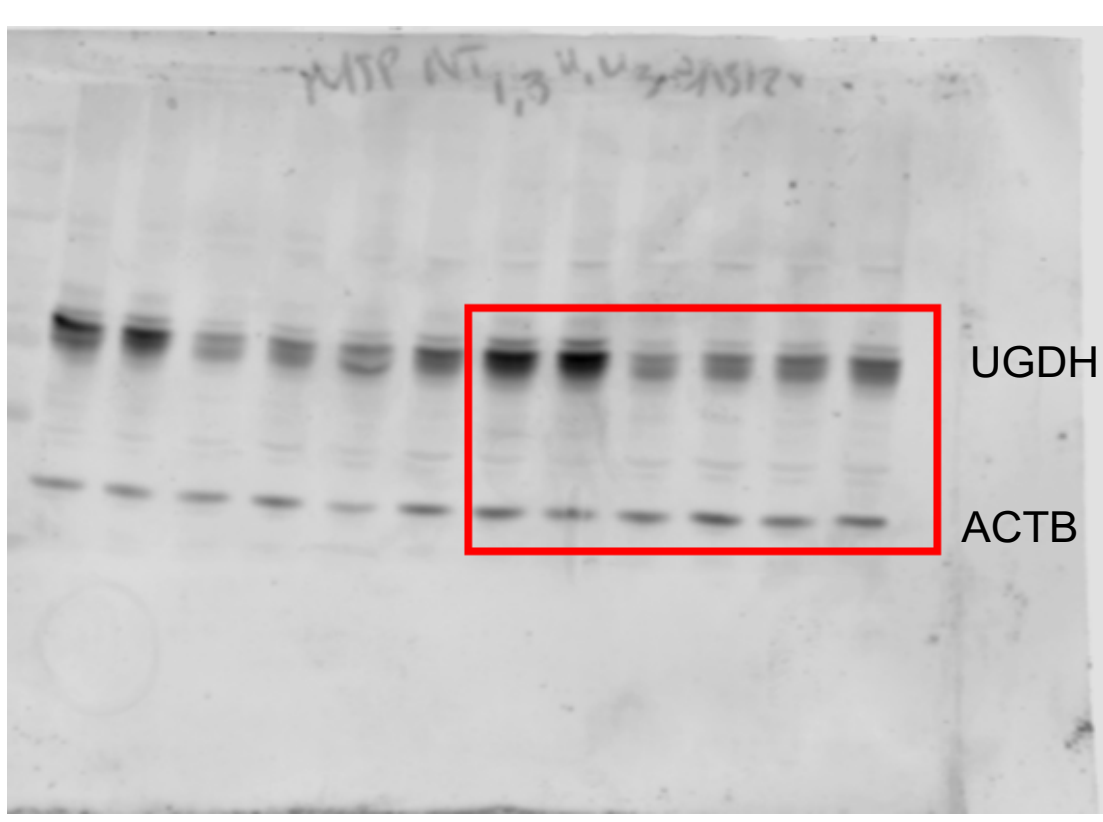

Figure 2C: MDA231 RNA values

Home

	NT shRNA MDA-MB-231	U1 shRNA MDA-MB-231	U2 shRNA MDA-MB-231
Veh	1.116260483	0.009018388	0.200092499
E2	1.133809746	0.013803286	0.102811033

Figure 4F: MCF7-GPER, + rUGDH
NT MCF7, U1 MCF7, U2 MCF7
G1, G15, G15+E2, G15+G1 tx
(UGDH bands from this blot)
ACTB from another blot (next page)

09.04.24 MCF7 GPER rUGDH GPER tx_GPR30 UGDH ACTB_BW.png

Figure 4C: MCF7-GPER, no rUGDH;
NT MCF7, U1 MCF7, U2 MCF7
G1, G15, G15+E2, G15+G1 tx
(UGDH and ACTB bands from this blot)

Home

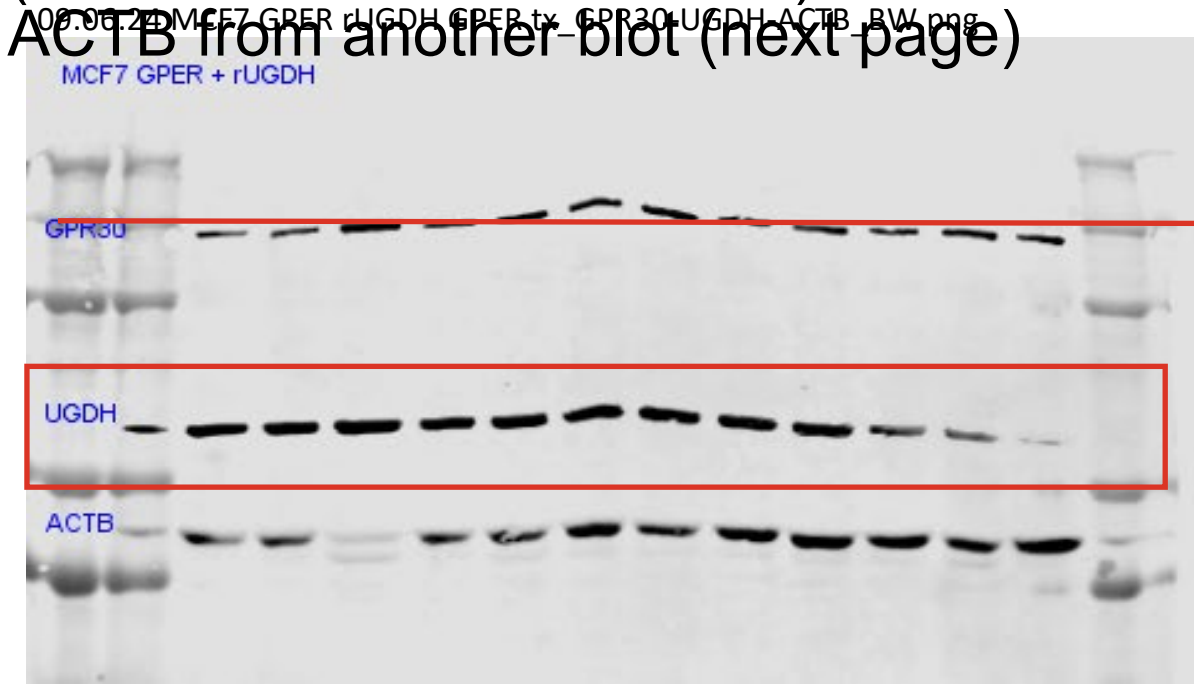

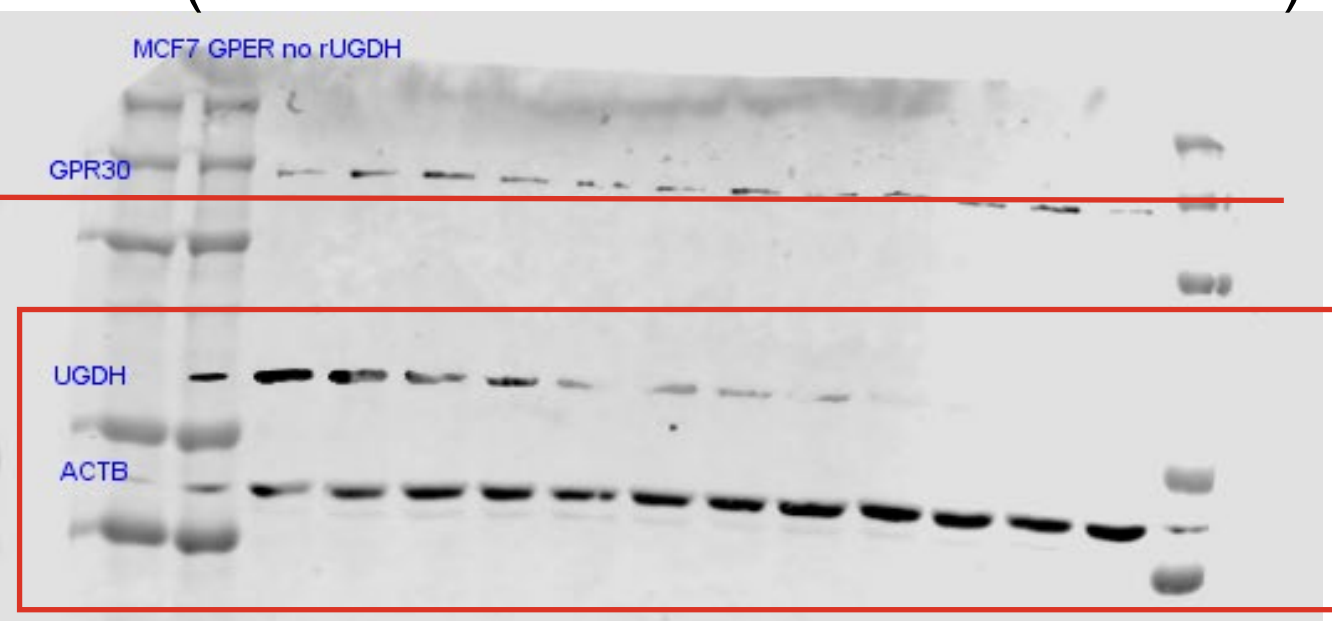

strikethru;
nonspecific bands

**repeated GPR30 and ACTB on another blot with same lysates to get better bands (for rUGDH blot and GPR30 for no rUGDH)
UGDH bands are from this blot and ACTB from no rUGDH are from this blot

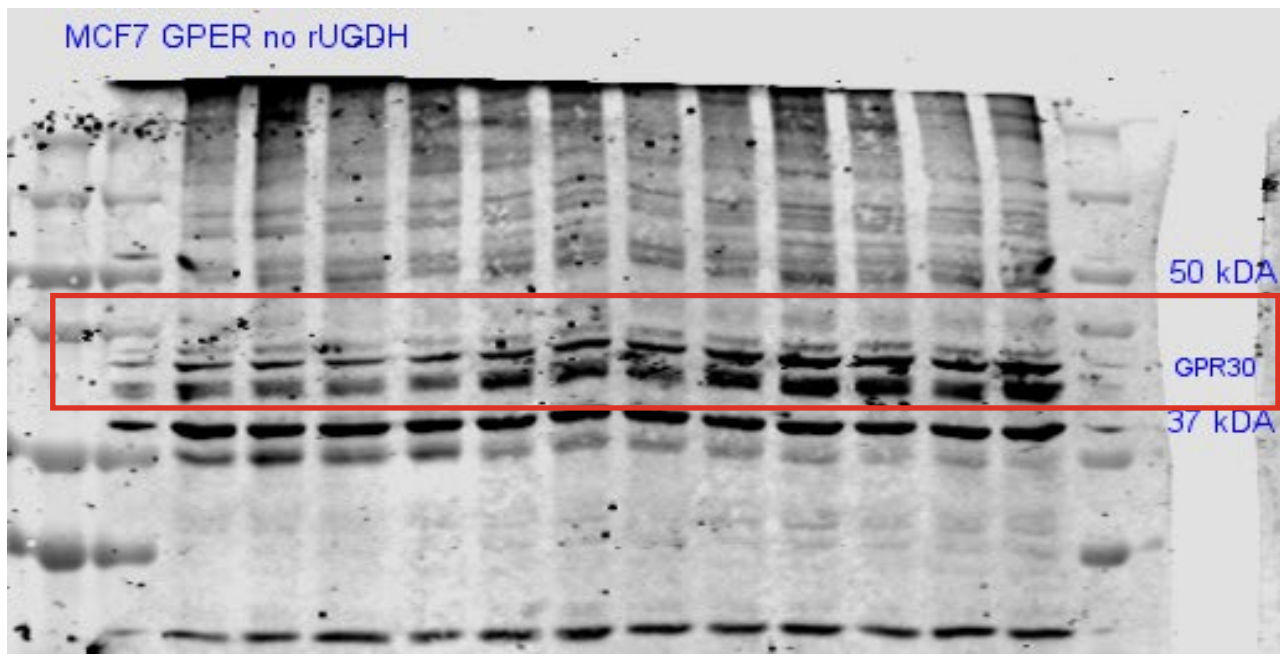

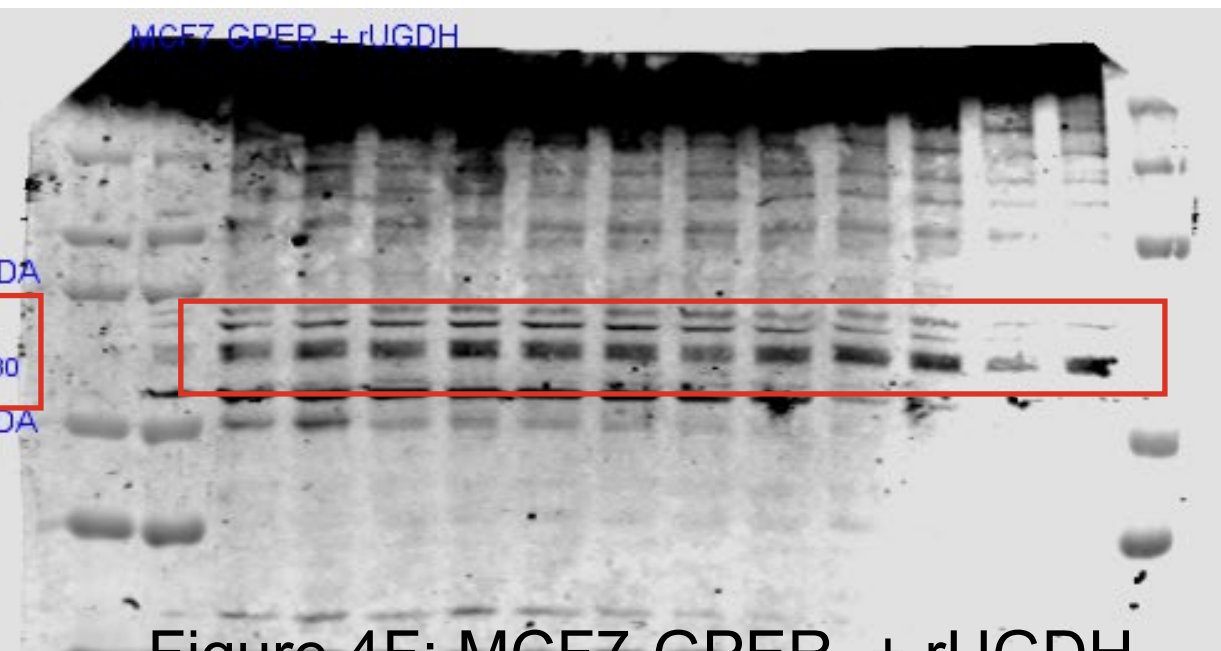

Figure 4F: MCF7-GPER, + rUGDH
NT MCF7, U1 MCF7, U2 MCF7
G1, G15, G15+E2, G15+G1 tx
(GPR30 bands from this blot)

**GPR30 bands from this blot
09.04.24 MCF7 GPER rUGDH GPER tx_GPR30 only BW.png

ACTB re-run on 9/24 --> find raw file

Figure 4C: MCF7-GPER, no rUGDH;
NT MCF7, U1 MCF7, U2 MCF7
G1, G15, G15+E2, G15+G1 tx
(GPR30 bands from this blot)

Figure 4C: MCF7-GPER, no rUGDH (as above)

09.06.24 MCF7 GPER rUGDH GPER tx GPR30-UGDH-ACTB BW.png

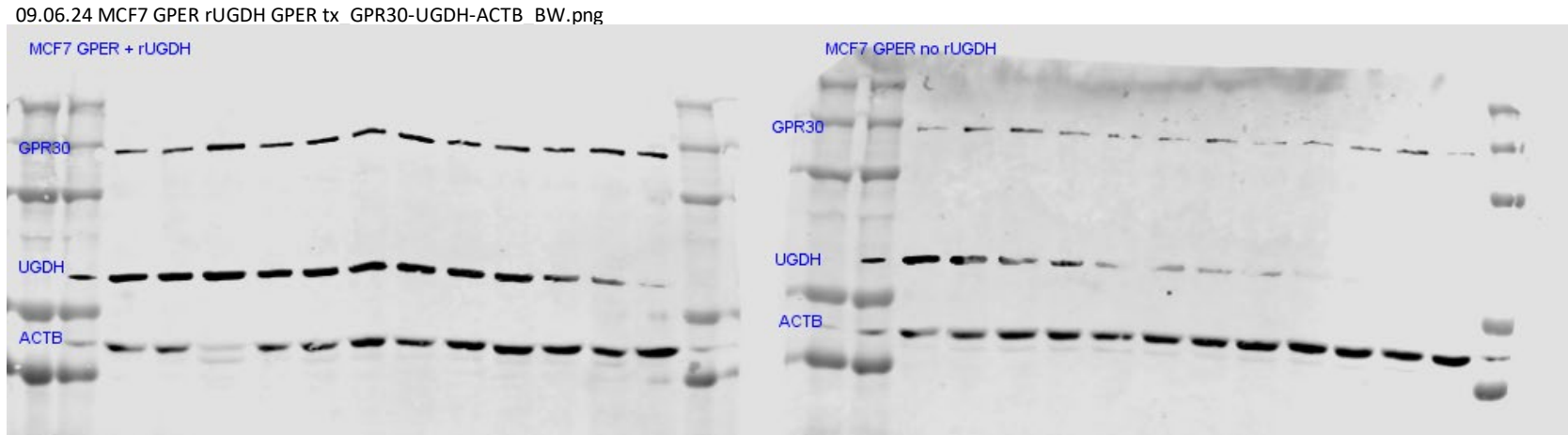

**repeated GPR30 and ACTB on another blot with same lysates to get better bands (for rUGDH blot and GPR30 for no rUGDH)
UGDH bands are from this blot and ACTB from no rUGDH are from this blot

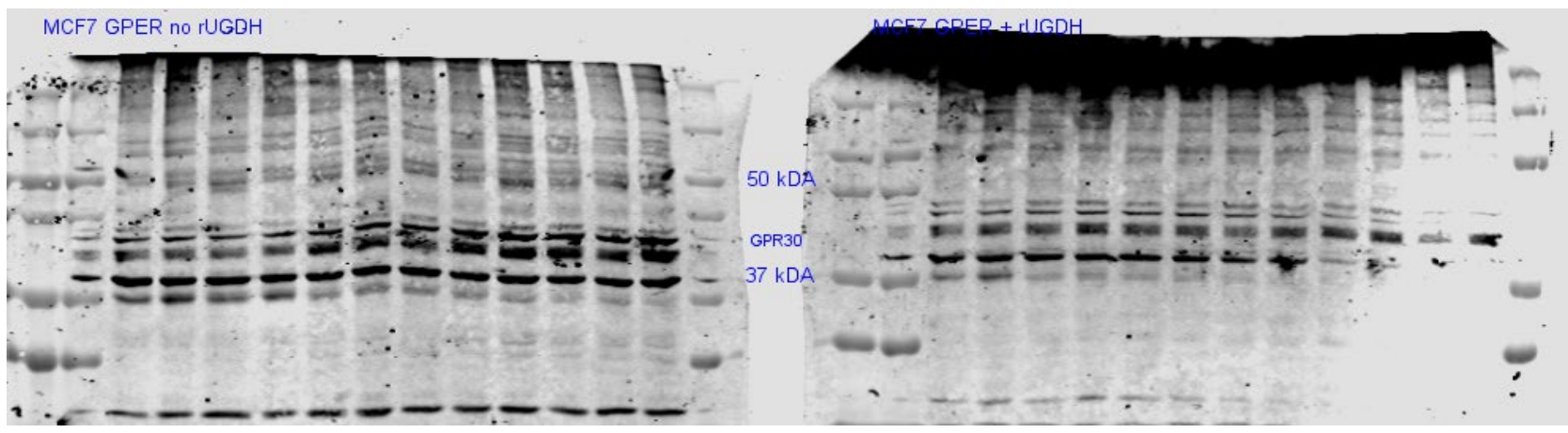

**GPR30 bands from this blot
09.04.24 MCF7 GPER rUGDH GPER tx_GPR30 only BW.png

ACTB re-run on 9/24 --> find raw file

Home

MCF7 GPER +/- rUGDH;GPR30 (r), UGDH (r), ACTB (m)

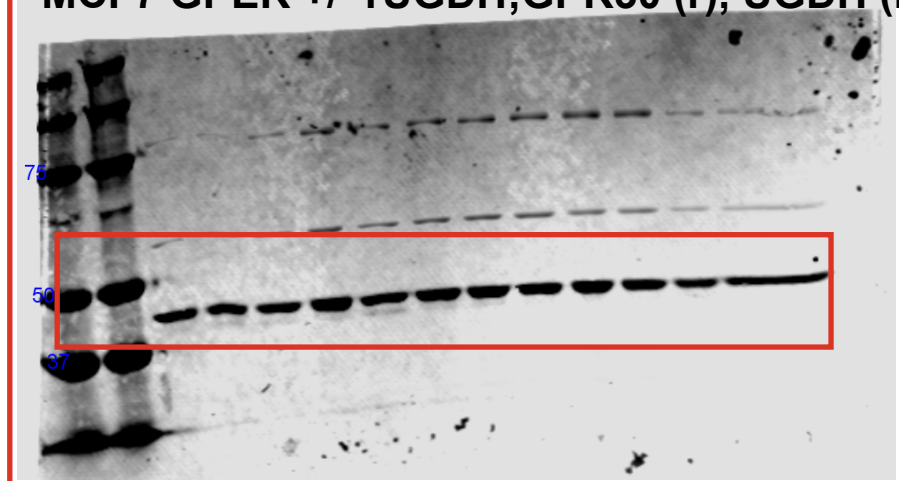

ACTB for Figure 4F

[Home](#)

no rUGDH rUGDH

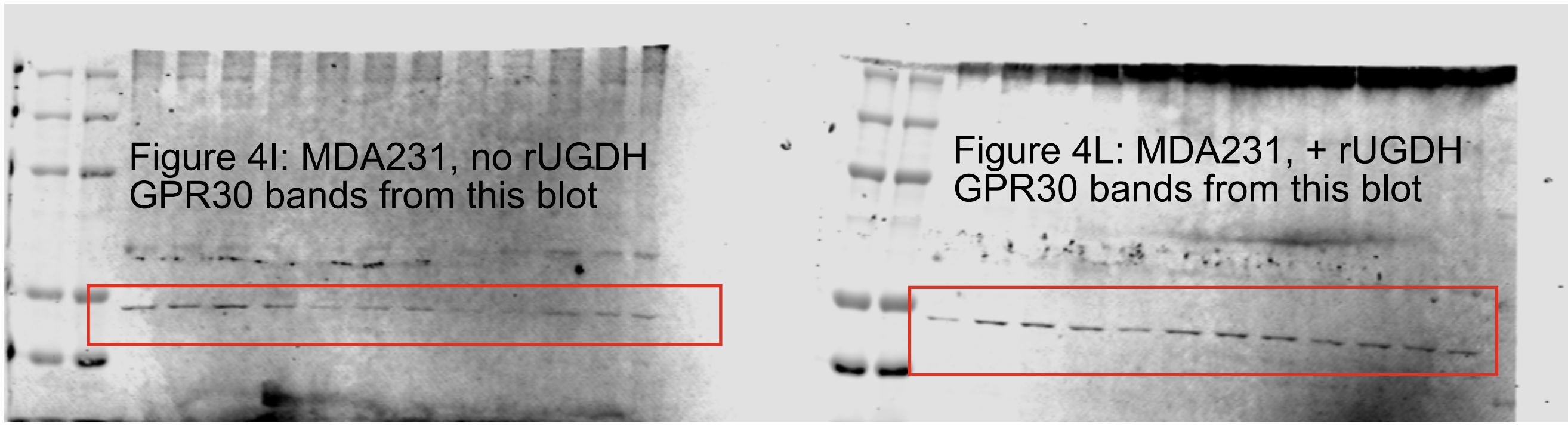

GPER is the bottom band
(in between 75kDA and 50kDA)

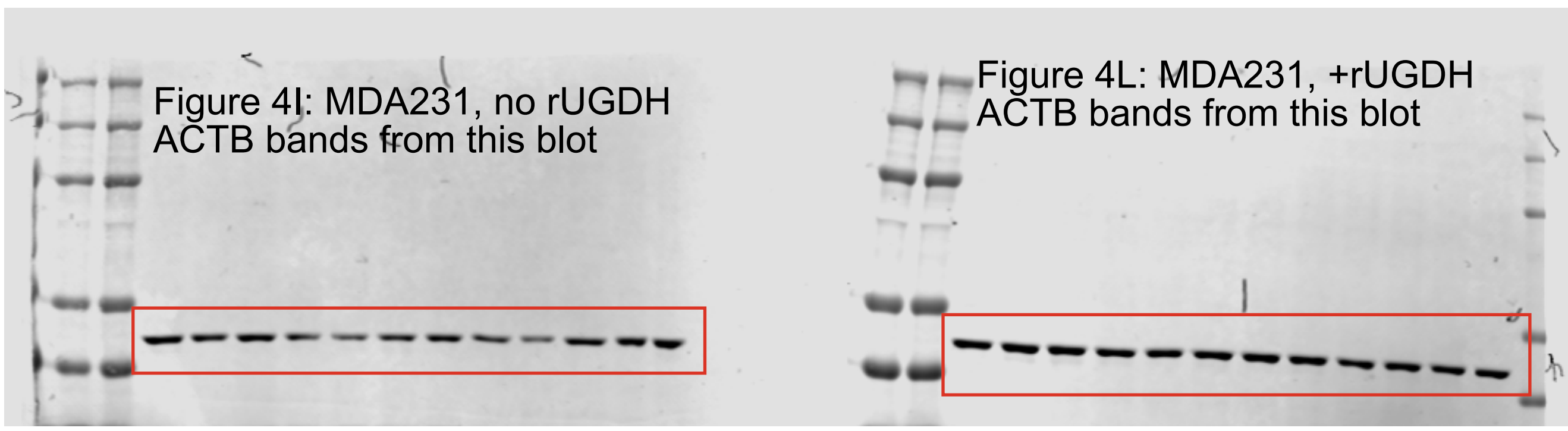

ACTB is this bottom band (10.21.24 MDA231 ACTB_GPERtx w-wo rUGDH BW)

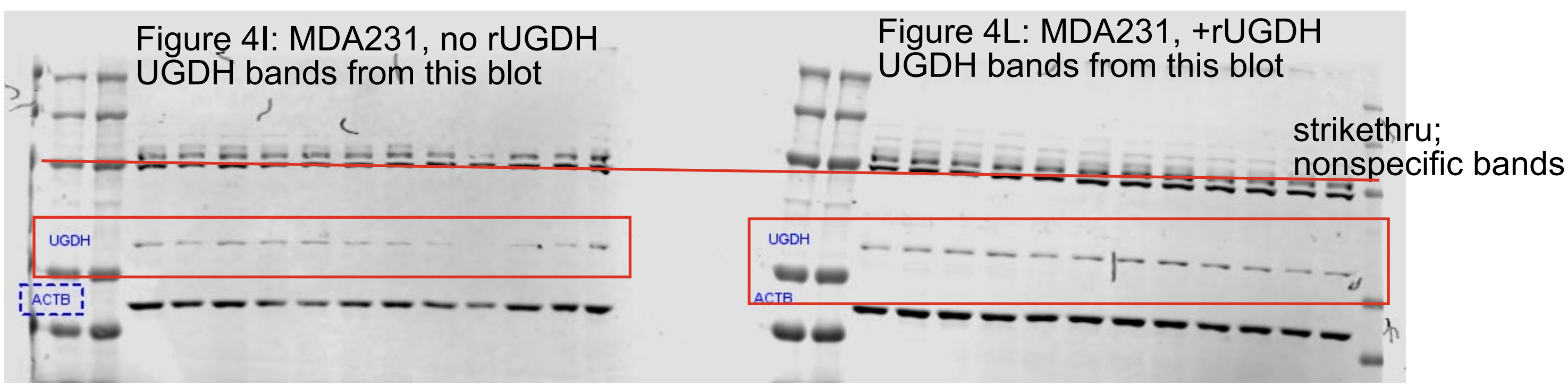

**nonspecific bands at 90-100 kDA

Figure 4I and Figure 4L Western Blots (this is a duplicate slide)

Home

no rUGDH rUGDH

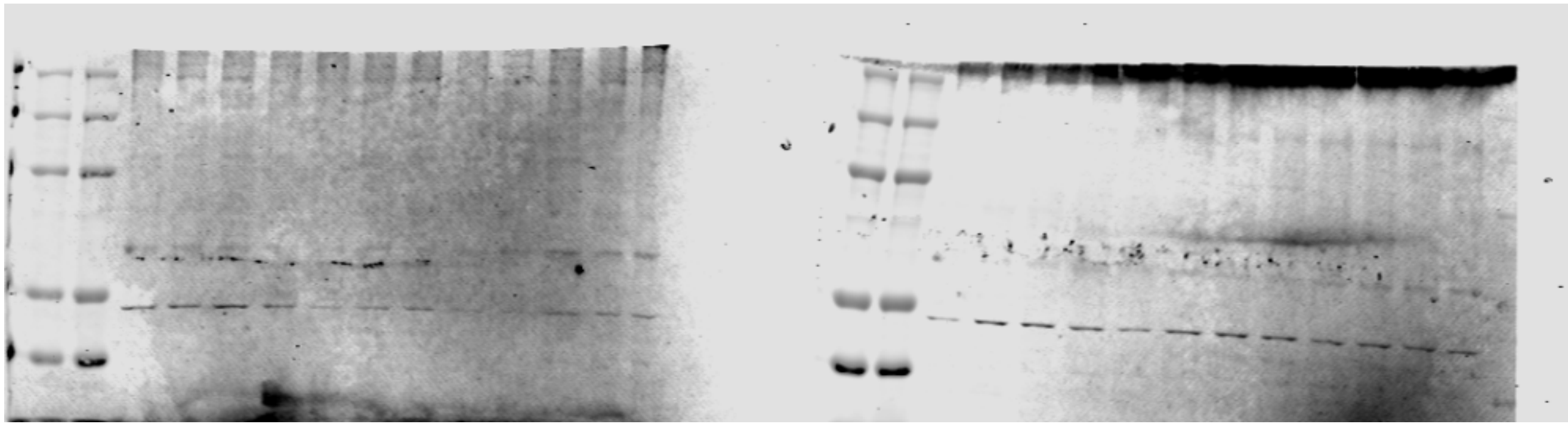

GPER is the bottom band
(in between 75kDA and 50kDA)

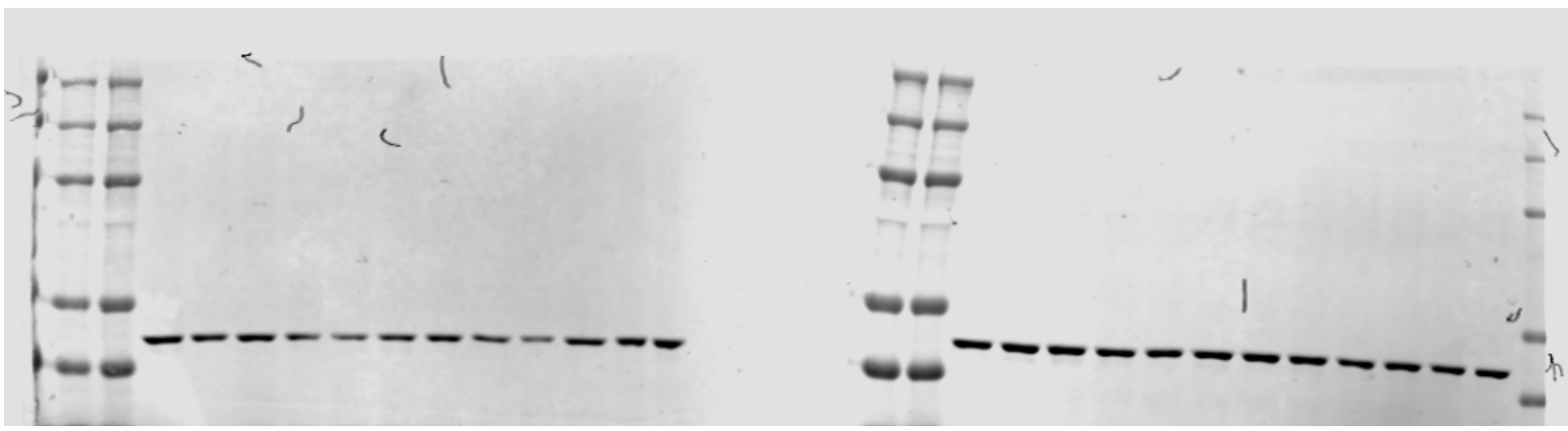

ACTB is this bottom band (10.21.24 MDA231 ACTB_GPERtx w-wo rUGDH BW)

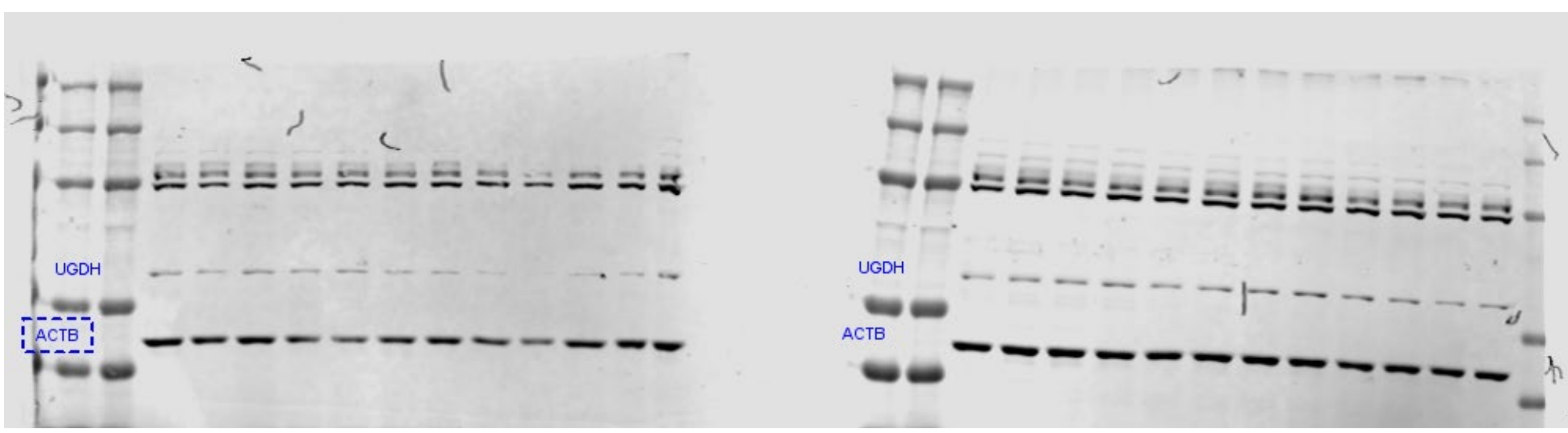

**nonspecific bands at 90-100 kDA

Supp. 1B_ER+ treated with estrogen for 24h (+) and 48h (++), cell lines UGDH expression

Home

MCF7			T47D			BT483			ZR-751			CAMA1		
-	+	++	-	+	++	-	+	++	-	+	++	-	+	++
5.5	6.8	6	1	1.25	1.65	3.25	3.1	3	1.9	1.85	1.75	1.85	2	1.5

Supp. 1C_ER negative breast cancer cell lines, UGDH protein expression

Home

NT MDA-MB-231	MDA-MB-231	MDA-MB-468	MDA-MB-436	MCF7 (+ control)
0.035718	0.023544	0.052273	0.056201	0.046843

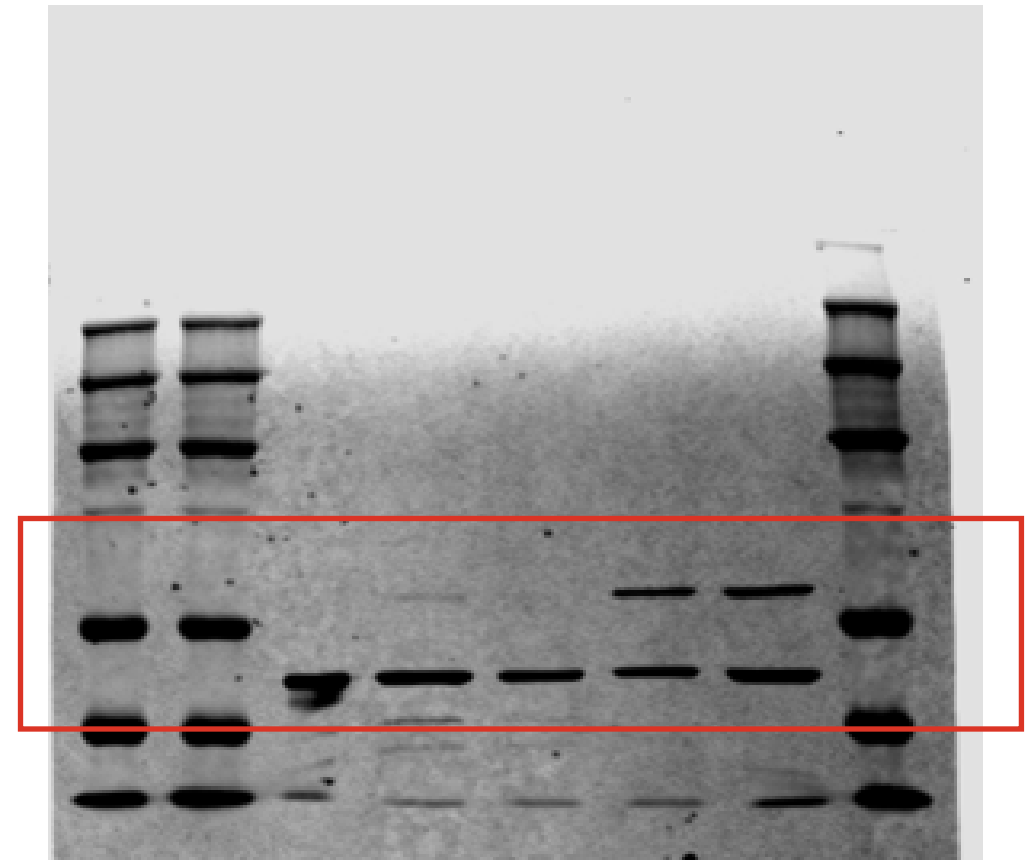

Supp. Fig. 1C: ER- breast cancer UGDH protein expression

Home

Supp. Fig. 4A: MCF7 GPER blot, raw image GPER and ACTB

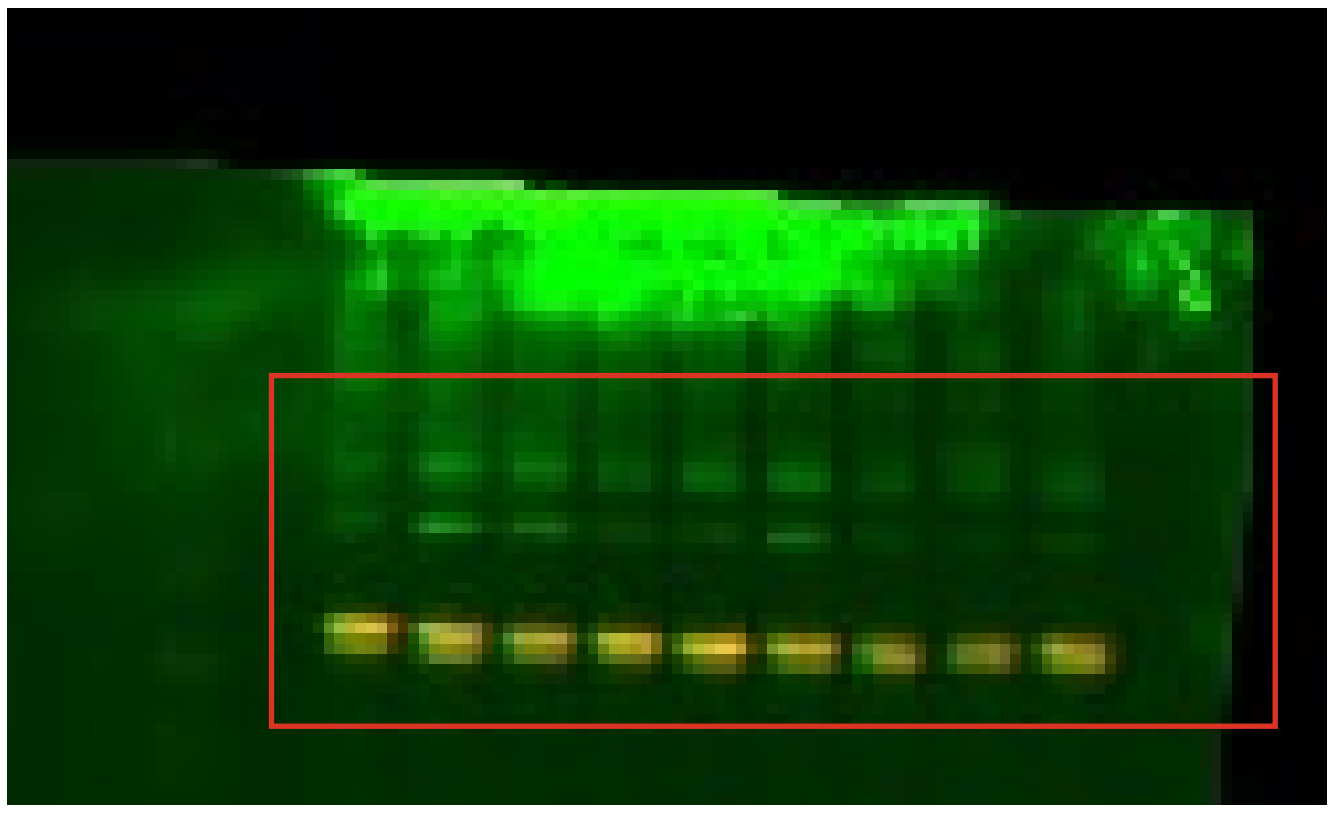

Home

Supp. Fig. 4B - MDA231 GPER blot

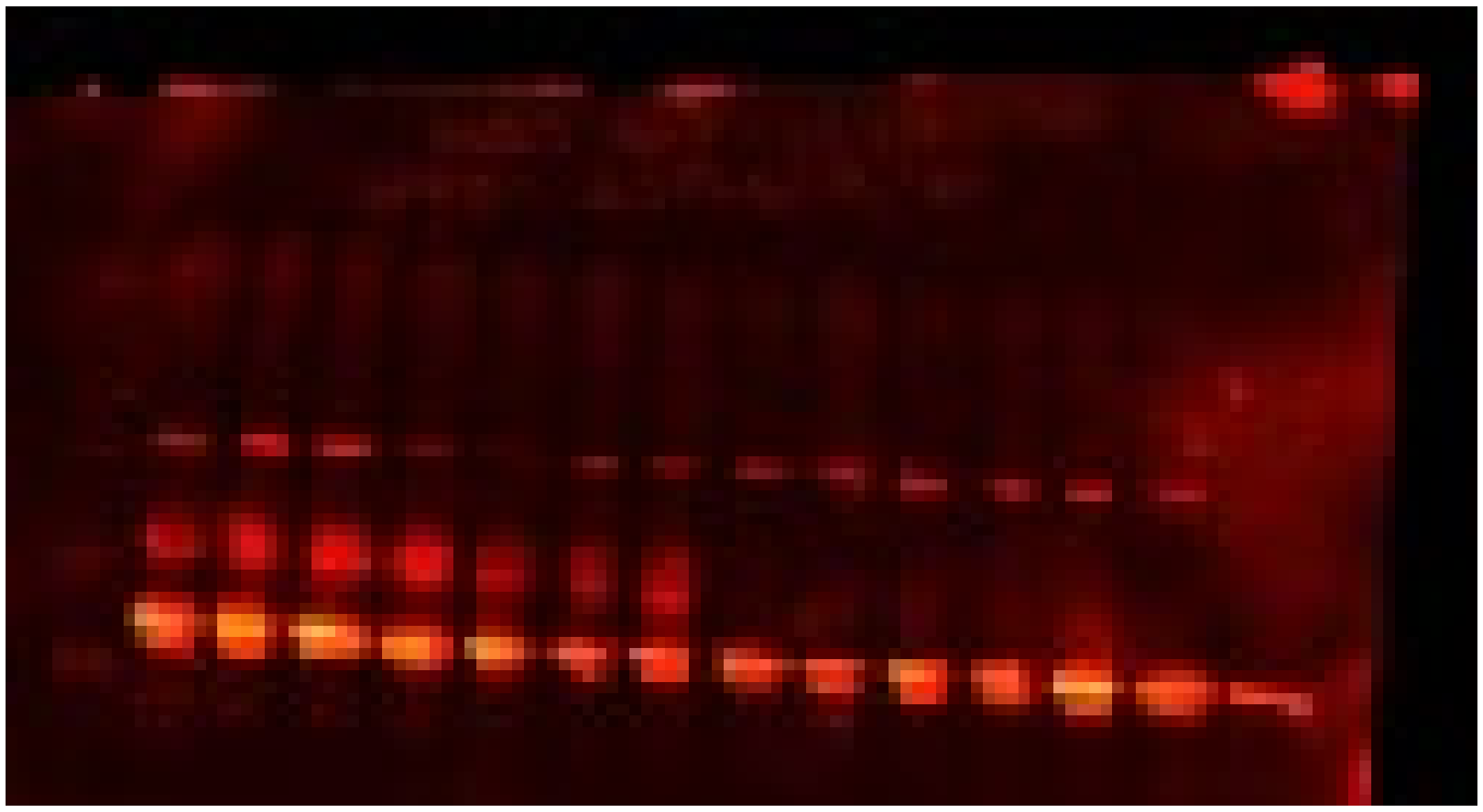

Supp. Fig. 5A_MDA231 mTOR blots

Home   Samples are the last four bands of each scanned blot: NT 231 + Veh, NT 231 + E2, U1 231 + Veh, and U1 231 + E2

ACTB

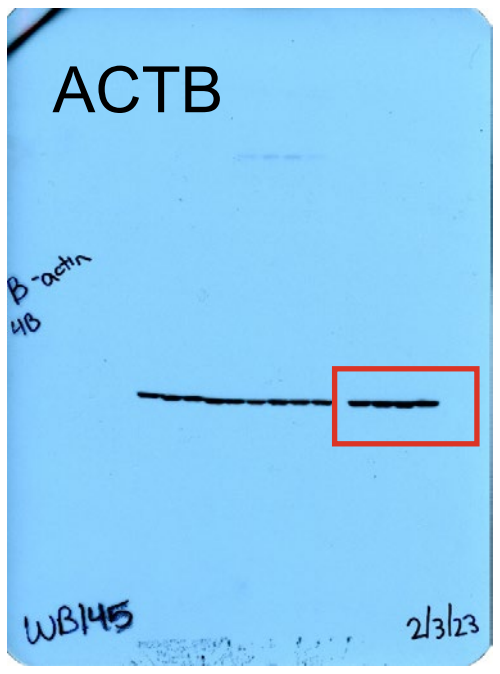

run 1- used E2F1 p4EBP1 from this

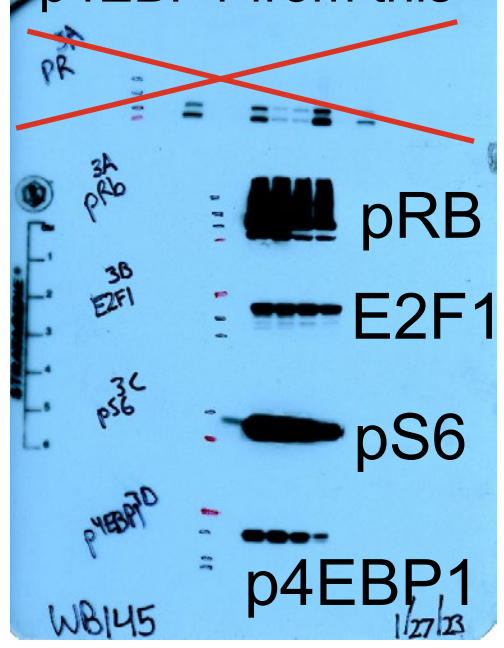

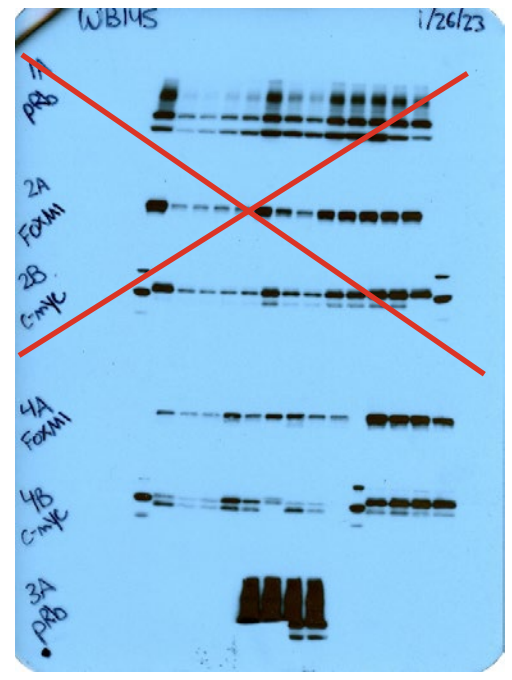

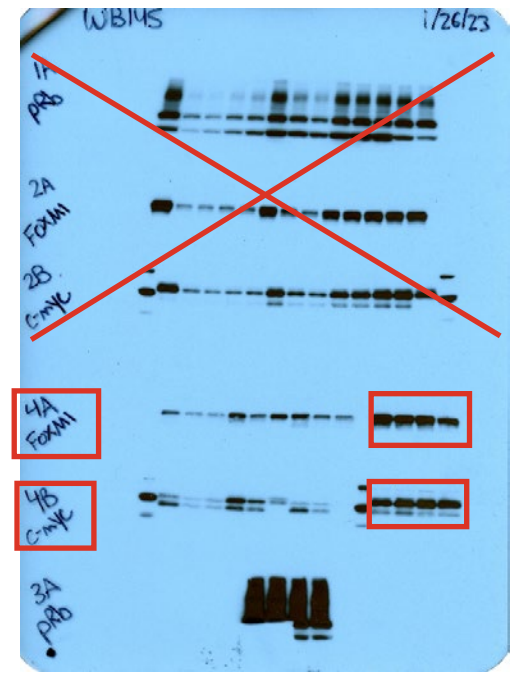

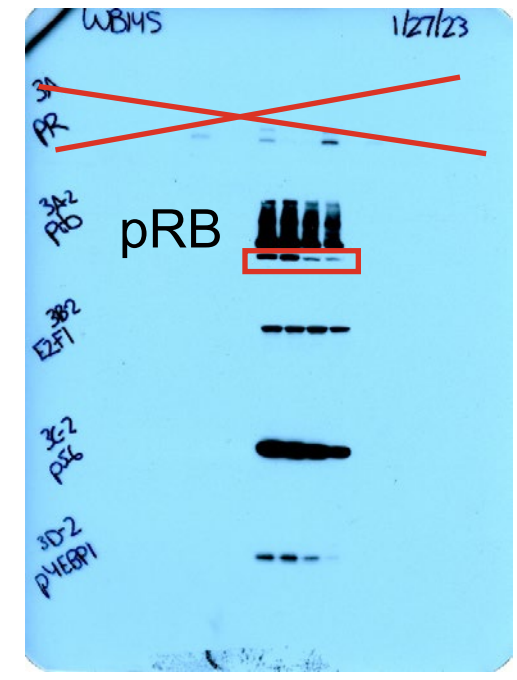

total 4EBP1

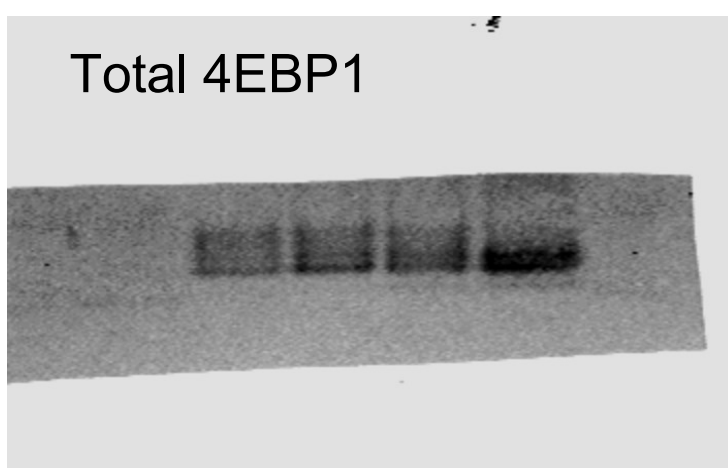

EGFR on top, IGFR on bottom

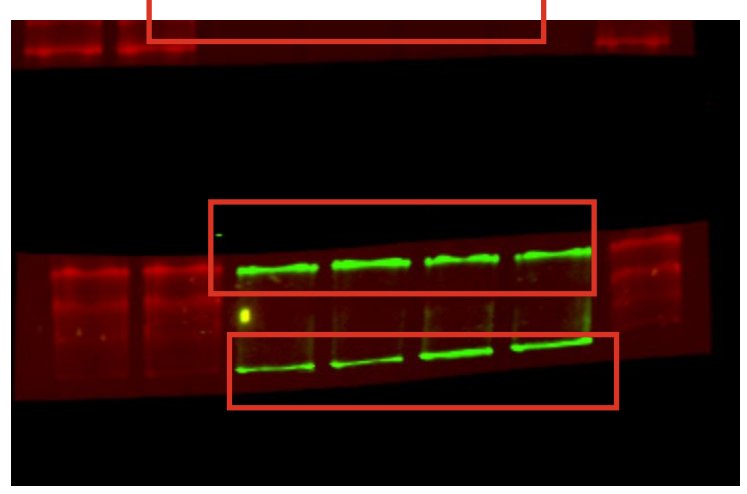

GPR30

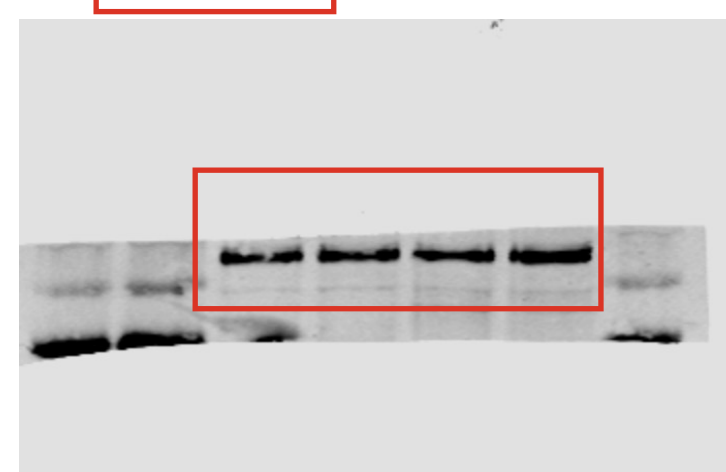

HER2

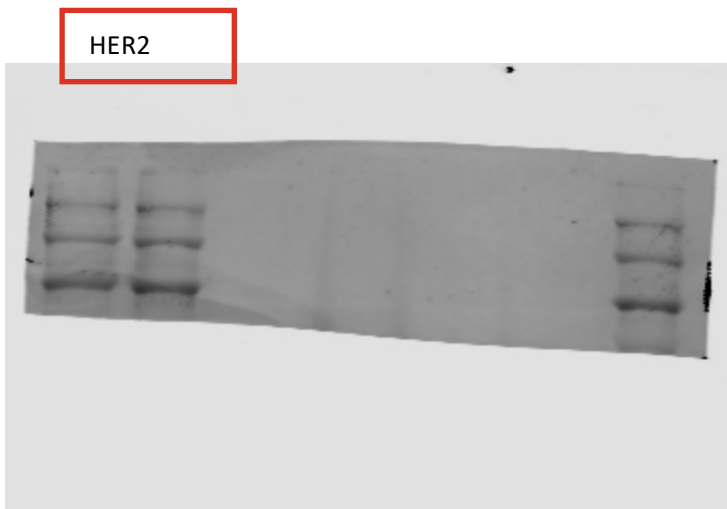

pAKT/ACTB

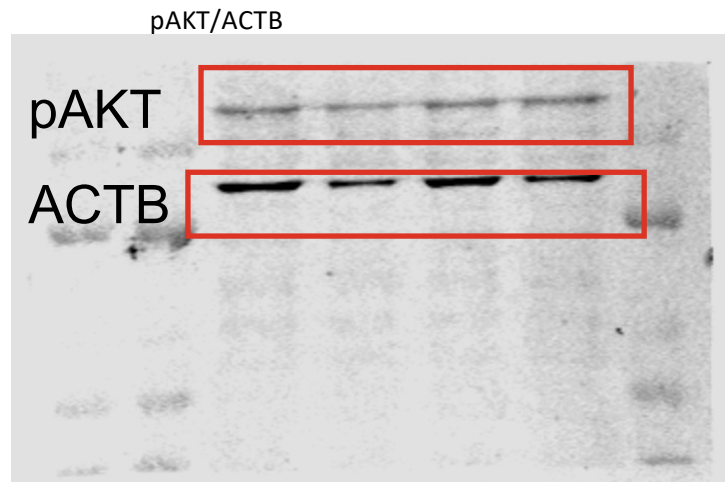

total RB

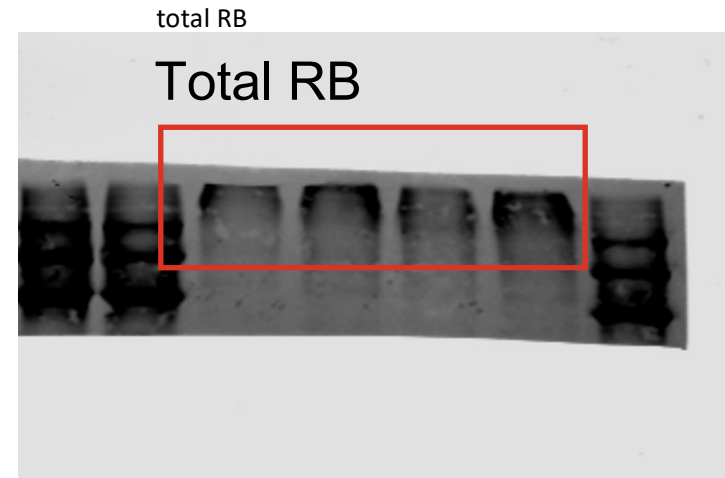

total S6

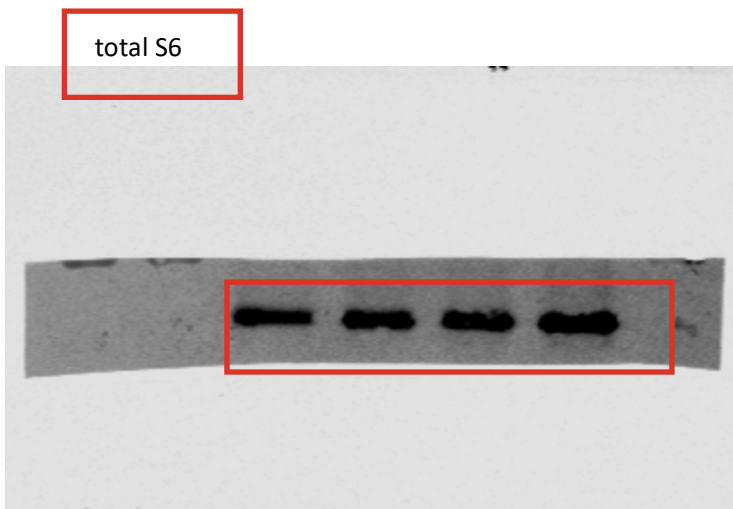

ER alpha - no bands where they were supposed to be
UGDH

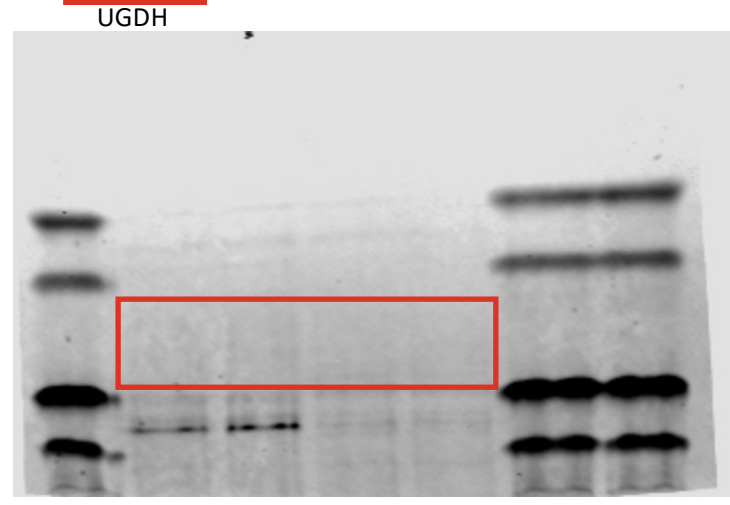

ERK

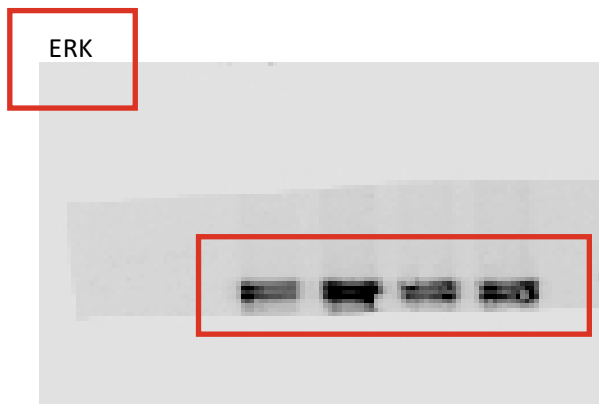

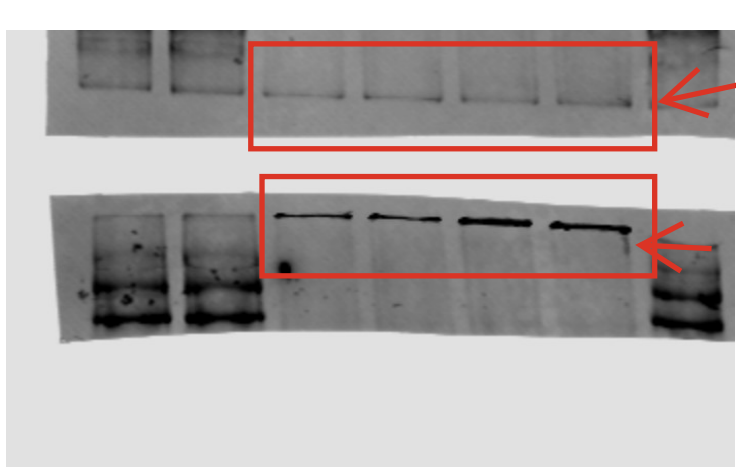

p-IGFRB

IGFRB

p-ERK

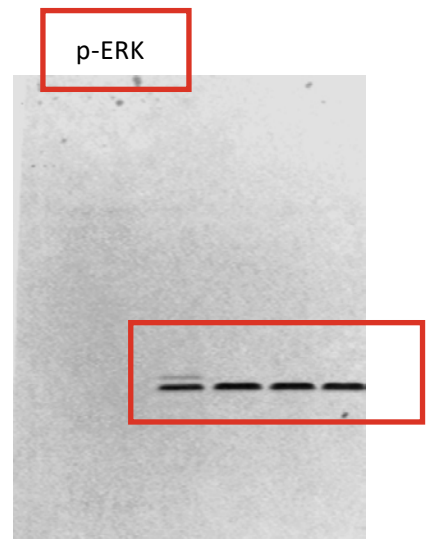

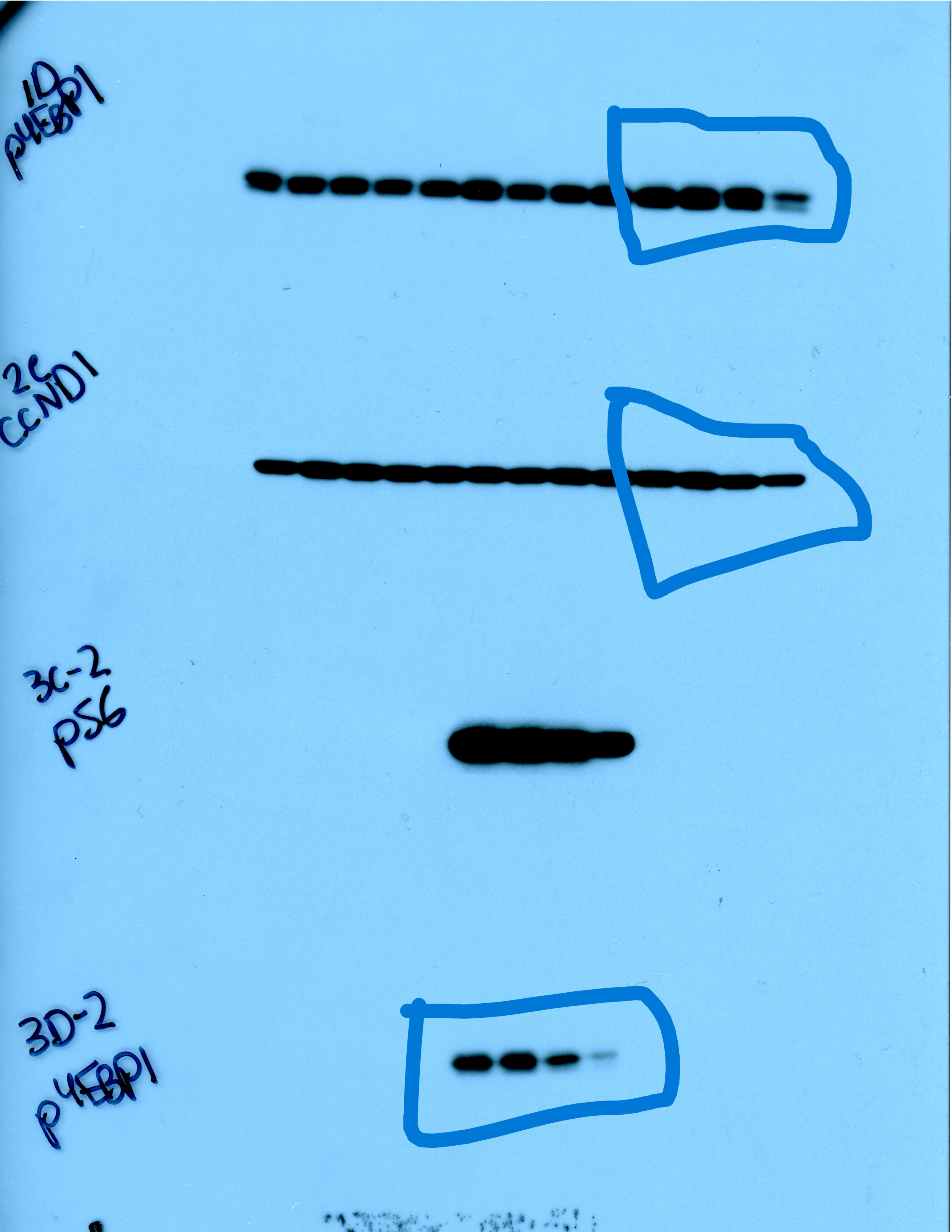
1D
p4EBP1
2C
CCND1
3C-2
PS6
3D-2
p4EBP1
